# Response and adaptation of the transcriptional heat shock response to pressure

**DOI:** 10.3389/fmicb.2024.1470617

**Published:** 2024-11-18

**Authors:** Carleton H. Coffin, Luke A. Fisher, Sara Crippen, Phoebe Demers, Douglas H. Bartlett, Catherine A. Royer

**Affiliations:** ^1^Graduate Program in Biochemistry and Biophysics, Rensselaer Polytechnic Institute, Troy, NY, United States; ^2^Marine Biology Research Division, Scripps Institution of Oceanography, University of California, San Diego, La Jolla, CA, United States; ^3^Department of Biological Sciences, Rensselaer Polytechnic Institute, Troy, NY, United States

**Keywords:** pressure, transcriptional heat shock response, number and brightness microscopy, adaptation, *E. coli*

## Abstract

**Introduction:**

The molecular mechanisms underlying pressure adaptation remain largely unexplored, despite their significance for understanding biological adaptation and improving sterilization methods in the food and beverage industry. The heat shock response leads to a global stabilization of the proteome. Prior research suggested that the heat shock regulon may exhibit a transcriptional response to high-pressure stress.

**Methods:**

In this study, we investigated the pressure-dependent heat shock response in *E. coli* strains using plasmid-borne green fluorescent protein (GFP) promoter fusions and fluorescence fluctuation microscopy.

**Results:**

We quantitatively confirm that key heat shock genes-*rpoH*, *rpoE*, *dnaK*, and *groEL* - are transcriptionally upregulated following pressure shock in both piezosensitive *Escherichia coli* and a more piezotolerant laboratory-evolved strain, AN62. Our quantitative imaging results provide the first single cell resolution measurements for both the heat shock and pressure shock transcriptional responses, revealing not only the magnitude of the responses, but also the biological variance involved. Moreover, our results demonstrate distinct responses in the pressure-adapted strain. Specifically, *P_groEL_* is upregulated more than *P_dnaK_* in AN62, while the reverse is true in the parental strain. Furthermore, unlike in the parental strain, the pressure-induced upregulation of *P_rpoE_* is highly stochastic in strain AN62, consistent with a strong feedback mechanism and suggesting that RpoE could act as a pressure sensor.

**Discussion:**

Despite its capacity to grow at pressures up to 62 MPa, the AN62 genome shows minimal mutations, with notable single nucleotide substitutions in genes of the transcriptionally important *b* subunit of RNA polymerase and the Rho terminator. In particular, the mutation in RNAP is one of a cluster of mutations known to confer rifampicin resistance to *E. coli* via modification of RNAP pausing and termination efficiency. The observed differences in the pressure and heat shock responses between the parental MG1655 strain and the pressure-adapted strain AN62 could arise in part from functional differences in their RNAP molecules.

## Introduction

In recent years it has become clear that a majority of microbial life exists in a diverse range of environments, most of which are inhospitable to humans (Merino et al., 2019). Among the characteristics possessed by organisms that thrive in the deep biosphere is piezotolerant or piezophilic growth; the ability to grow or preferentially grow, respectively, at high hydrostatic or lithostatic pressures. In addition to piezotolerant/philic adaptation to grow under high pressure, mesophiles can acquire pressure resistance to survive brief but large pressure shocks (Malone et al., 2006; Van Boeijen et al., 2010; Vanlint et al., 2011, 2012). This process poses a major problem for high pressure processing (HPP) of foods, which is a multibillion-dollar industry projected to grow significantly over time as pressure treatment, unlike temperature sterilization, allows for the retention of food taste and texture (Huang et al., 2017). Beyond mere resistance to pressure, *E. coli* has been observed to acquire the ability to grow under high pressure in a laboratory setting (Marietou et al., 2015). In this study, adaptive laboratory evolution (ALE) was used to develop *E. coli* strain AN62, which is capable of growth up to 62 MPa. Only 17 mutations were found in the genome of AN62 (Supplementary Table S1; Allemann et al., 2024).

All cellular components respond to increasing pressure (Bartlett, 2002; Oger and Jebbar, 2010; Gayán et al., 2017). Beyond effects on individual molecules, pressure leads to increased activity of promoters recognized by the general stress response sigma factor RpoS (σ^S^), which has been implicated in pressure resistance (Vanlint et al., 2013b). Notably, sub-lethal pressure shock has been shown to elicit the upregulation of numerous *E. coli* heat shock proteins (HSPs), including DnaK and GroEL (Welch et al., 1993), as well as the transcriptional induction of HSP genes post sub-lethal pressure shock (Aertsen et al., 2004). Upregulation of HSPs also leads to improved bacterial survival during a lethal pressure shock (Aertsen et al., 2004). The gene for the heat shock regulated extra-cytoplasmic stress response sigma factor *rpoE* (produces σ^E^ or RpoE) is also transcriptionally induced and enhances viability following lethal pressure exposure (Malone et al., 2006). Similar examinations of pressure-induced transcriptional heat shock responses, as well as observations of cross resistance between heat and pressure shocks have reinforced the hypothesis that the heat shock response is important for high pressure adaptation and survival and underscores the importance of proteostasis for bacterial survival and growth under stress (Aertsen and Michiels, 2007; Vanlint et al., 2013a; Gayán et al., 2016).

The ultimate outcome of the heat shock response is to upregulate two key groups of HSPs: the DnaK/DnaJ and GroEL/GroES chaperone systems (Saito and Uchida, 1978; Kusukawa and Yura, 1988; Lipinska et al., 1988). When the proteome is destabilized, DnaK works in tandem with its co-transcribed chaperone, DnaJ, and a nucleotide exchange factor, GrpE, as an unfoldase to disaggregate and partially unfold misfolded or aggregated proteins (Slepenkov and Witt, 2002). In contrast, under homeostatic conditions DnaK sequesters RpoH (σ^32^), the main heat shock sigma factor, thereby repressing its transcriptional activation activity (Johnson et al., 1989; Straus et al., 1990; Gamer et al., 1992; Gamer et al., 1996). It has also been shown that numerous proteins require DnaK to fold properly or maintain their proper folding (Calloni et al., 2012). GroEL functions as a large multimeric complex with GroES and is required for the folding of several important proteins defined as class IV substrates (Fujiwara et al., 2010) and to refold unfolded or misfolded proteins (Kerner et al., 2005). It has been proposed that DnaK may act as a filter for GroEL selectivity (Kerner et al., 2005; Calloni et al., 2012).

**Figure 1:**
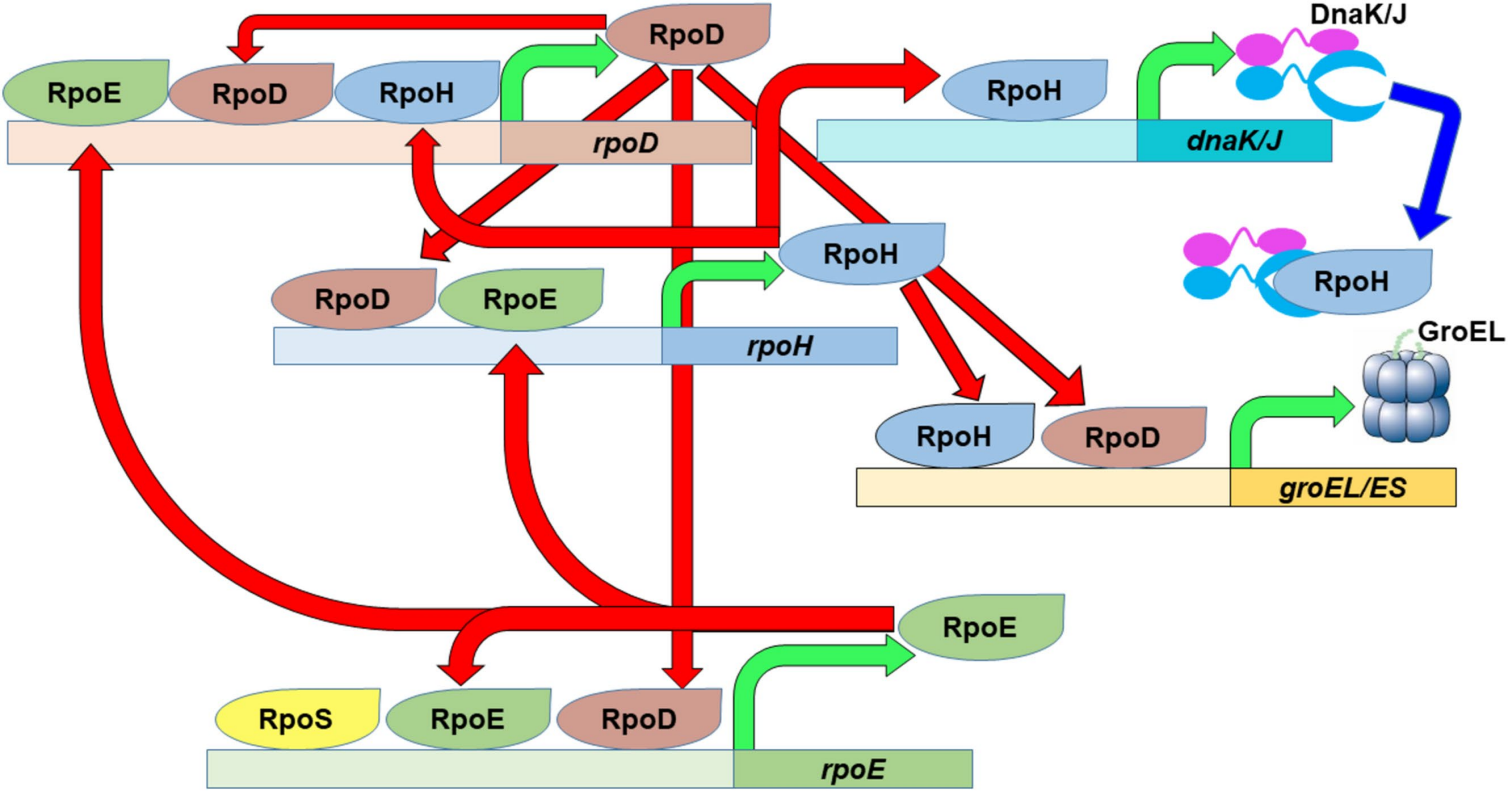
The heat shock response in *E. coli*. The main housekeeping sigma factor, RpoD, as well as the primary (RpoH) and secondary (RpoE) heat shock sigma factors possess complex promoter regions that allow them to fine tune their expression based on the needs of the cell. An increase in the amount of RpoH will eventually lead to increased expression of specific chaperon systems (DnaK/J and GroEL/ES) in order to stabilize the proteome after temperature upshift. Green arrows depict transcription of the designated gene to produce the specified protein product. Red arrows depict the transcriptional activation activity of the specified sigma factors. Blue arrow depict depicts repression activity of the specified chaperones.

The heat shock response is heavily regulated, particularly at the transcriptional level via the alteration of utilized sigma factors and promoters in HSP gene promoter regions (Figure 1). Thermal induction of HSP genes, including *dnaK/dnaJ* and *groEL/groES* (Cowing et al., 1985; Cowing and Gross, 1989), is achieved by a large increase in the quantity of the heat shock sigma factor RpoH (Grossman et al., 1987), a normally very unstable protein (Tilly et al., 1989). The promoter region of the *rpoH* gene is complex, allowing its expression to be driven by either the main sigma factor RpoD (σ^70^) or by RpoE (σ^E^) (Erickson et al., 1987; Wang and Kaguni, 1989a), a secondary heat shock sigma factor (Rouviere et al., 1995). The regulation of RpoE at the transcriptional and post translational levels depends on changes in the amount of unfolded proteins; particularly those associated with the cell membrane and periplasm (Raina et al., 1995; Missiakas et al., 1997). Like that of *rpoH*, the *rpoE* promoter region is controlled by multiple sigma factors, RpoD, RpoS and RpoE, along with sigma factors unrelated to the heat shock response (Klein et al., 2016). It is important to note that *rpoD* also experiences a heat shock response, whereby its expression can be driven by RpoE or RpoH in addition to RpoD (Burton et al., 1983; Taylor et al., 1984; Grossman et al., 1985). Note that other transcription factors, unrelated to the heat shock response and not shown in Figure 1, also contribute to the regulation of alternative sigma factor and HSP transcriptional regulation (Wang and Kaguni, 1989b; Kallipolitis and Valentin-Hansen, 1998; Landini et al., 2014; Klein et al., 2016; Ishihama, 2017; Rome et al., 2018).

In the present study, we sought to confirm and quantitatively characterize, at single cell resolution, the pressure-induced transcriptional heat shock response in *E. coli*. To this end we generated plasmid borne green fluorescent protein (GFP) promoter fusion constructs of four key heat shock genes: those encoding the chaperones, *dnaK* and *groEL*, and the two alternative sigma factors, *rpoE* and *rpoH*. We then quantified the transcriptional response of each promoter to heat and pressure shock in both the *E. coli* K-12 strain MG1655 (Blattner et al., 1997) and its derived high pressure-adapted strain, AN62 (Marietou et al., 2015; Allemann et al., 2024). Quantification of the absolute GFP concentration in single cells prior to and after heat or pressure shock was carried out using a particle counting imaging approach called two photon scanning number and brightness microscopy (sN&B), which was specifically designed to perform quantitative measurements in live cells with minimal photobleaching, low background fluorescence, single cell resolution, and the ability to differentiate between an increase in the number of cells vs. an increase in the fluorescence intensity per cell (Digman et al., 2008; Ferguson et al., 2011, 2012; Royer, 2019). Our results confirmed that *E. coli* mounts a heat shock response at the transcriptional level when exposed to pressure shock, and that for some promoters the response to pressure shock differs in magnitude from the response to heat shock. We also found that upregulation of *P_rpoH_* was consistently larger after pressure shock compared to heat shock in both MG1655 and AN62, which underscores the importance of the pressure-induced heat shock response, even for organisms that can grow under high pressure. Finally, we show that the transcriptional pressure shock response is distinct for the chaperone genes *dnaK* and *groEL* in *E. coli* MG1655 and AN62. Pressure-induced *dnaK* upregulation is stronger in MG1655, while that of *groEL* is more pronounced in AN62. These observations suggest that producing more GroEL than DnaK might provide a selective advantage for growth under pressure.

## Materials and methods

### Strain construction

GFP transcriptional fusions were constructed for four major heat shock genes: the chaperone-encoding *dnaK* and *groEL* genes, and the alternate *σ* factor-encoding *rpoE* and *rpoH* genes. They were cloned into plasmid pMS201, which is maintained as a low copy number plasmid (Zaslaver et al., 2006; Table 1). For each reporter fusion, the full-length promoter region, encompassing all known promoters for each gene, was utilized (hereafter referred to as promoter fusion for simplicity). All plasmids were purchased from Horizon Discovery and transformed into *E. coli* K12 strains MG1655 or AN62. Cells were made chemically competent via the Transformation Storage Solution (TSS) method (Chung and Miller, 1993). pMS201 utilizes 50 μg/mL kanamycin for plasmid selection.

**Table 1:** Relevant strains and plasmids used in this study.

Strain/Plasmid	Relevant characteristics	Source
*E. coli* MG1655	Wild type *E. coli*, source of promoters for pMS201 plasmids	The Coli genetics stock center (CGSC)
*E. coli* AN62	High pressure-adapted strain derived from MG1655	Marietou et al. (2015)
pMS201-*P_dnaK_*::GFP	Full length *dnaK* promoter region transcriptionally fused to the GFPmut2 gene	Horizon discovery
pMS201-*P_groEL_*::GFP	Full length *groEL* promoter region transcriptionally fused to the GFPmut2 gene	
pMS201-*P_rpoE_*::GFP	Full length *rpoE* promoter region transcriptionally fused to the GFPmut2 gene	
pMS201-*P_rpoH_*::GFP	Full length *rpoH* promoter region transcriptionally fused to the GFPmut2 gene	
*E. coli* MG1655 *P_BAD_-gfp-mrr*	Full length arabinose inducible promoter region transcriptionally fused to free GFP and unlabeled Mrr	Bourges et al. (2017)
pBAD24-*P_BAD_*::GFP	Full length arabinose inducible promoter region transcriptionally fused to the GFPmut2 gene	This study

An exception was the transcriptional fusion of GFPmut2 being driven by the arabinose inducible promoter *P_BAD._* It was generated via Gibson assembly using the plasmid pBAD24 as a backbone and GFPmut2 as the insert and transformed into *E. coli* strain MG1655. Unlike pMS201, pBAD24 utilizes 100 μg/mL ampicillin for selection.

The next day, transformants were re-streaked onto selective plates containing the necessary antibiotic and verified by PCR. Clonal isolates verified by PCR were grown to mid-upper log phase and 1 mL of culture was preserved in 25% (v/v) glycerol and stored at −80°C. Plasmid sequence integrity was also verified via whole plasmid sequencing (Primordium Labs) (Supplementary Table S2) after being harvested from 1 mL of mid-upper log phase cultures from clonal isolates using a Zymo Research ZR-Plasmid Miniprep™-Classic kit. Unless otherwise stated, all culturing and recovery steps were done in Luria Broth (LB) containing per liter 10 g Tryptone, 10 g sodium chloride, and 5 g yeast extract supplemented with the correct antibiotic for plasmid selection.

### Cell culture

*E. coli* AN62 and its mesophilic ancestor MG1655 were used for sN&B experiments. Unless otherwise stated, all culturing was done in LB medium supplemented with 50 μg/mL kanamycin. For heat shock experiments, cells were grown at 30°C at 180 rpm. For pressure shock experiments, cells were grown at 37°C at 180 rpm to decrease the likelihood of high-pressure inactivation (Aertsen et al., 2004). After overnight growth, MG1655 cells were diluted 1:100 fresh and AN62 cells were diluted 1:10 into medium. AN62 cells were diluted significantly less than MG1655 to skip their long lag phase (Marietou et al., 2015). All cultures were allowed to grow to mid-log phase (OD_600 nm_ = 0.4–0.5). At mid log phase, two aliquots of 600 μL of cells were removed from the culture. One aliquot was prepared for imaging without any shock, while the other aliquot was subjected to either a heat or pressure shock. We verified balanced growth conditions, as results were similar when cells were grown after a 1:10,000-fold dilution.

### Heat and pressure shocks

For heat shock, cells were placed in a 42°C water bath for 15 min and then prepared for imaging. For pressure shock, cells were transferred to a quartz cuvette and sealed with a DuraSeal cap and an O-ring. The cuvette was then placed inside a high-pressure cell and pressurized to 60 MPa (600 bar). The setup for the high-pressure cell has been previously described (Jenkins et al., 2018). The pressurization was performed in increments of 20 MPa (200 bar), with a brief equilibration period of 5 s at each pressure. Cells were pressurized for 15 min and kept at 34°C (The limit of the temperature regulation unit attached to the high-pressure cell). Depressurization was performed in the same manner as pressurization. After pressure shock, cells were transferred from the cuvette to a sterile Eppendorf tube and prepared for imaging.

### Cell preparation for imaging

Both aliquots were prepared for imaging using an agarose pad setup that has been previously described (Ferguson et al., 2011; Supplemental methods) with modifications. Briefly, cells were centrifuged at 7000 rpm for 2 min and resuspended in 3–5 μL of minimal M9 medium supplemented with 0.4% glucose (Supplementary Table S3). Cells were then plated on a 66 μL, 2% agarose pad made with the same supplemented M9 medium. Cells were allowed to equilibrate on the surface of the pad for 5 min, then a poly lysine coated No. 1 coverslip (VWR) was placed over top of the cells for 1 min before sealing the cells inside. The cells were then placed in an autofluor holder for imaging.

Because of the short time frame (under 10 min) between the end of the shock and mounting on the microscope, it is extremely unlikely that there was any significant amount of growth of the cells. This prevented any significant loss of GFP due to dilution from cell division. It is also unlikely there was any significant protease degradation since GFP has been observed to possess a long half-life in cells (Tombolini et al., 2006). This timeline also allows for rapid measurement of the response that occurred during or immediately after the shock and avoids any pleotropic effects due to differences in growth rates between MG1655 and AN62.

### Two photon excitation fluorescence fluctuation microscopy

Imaging was performed on an ISS Alba fast scanning mirror fluctuation microscope (ISS, Champaign, IL) equipped with 2-photon laser excitation (Mai Tai Ti: Sapphire, Newport-SpectraPhysics, Mountain View, CA). 930 nm excitation light (with an average power of 15.2 mW) was focused through a 60 × 1.2NA water immersion objective (Nikon APO VC) onto a No. 1 coverslip. All images were 20 μm x 20 μm. A 735 nm low-pass dichroic filter (Chroma Technology Corporation, Rockingham, VT, USA) was used to filter infrared light from the emitted light. Emitted light was further filtered with a 530/43 nm bandpass filter just before reaching the detector - an avalanche photodiode (Perkin Elmer). At the start of each experiment, 28 nM fluorescein was used to assess the quality of the laser alignment through Fluorescence Correlation Spectroscopy (FCS) and by determining the effective volume of the 2-photon point spread function (PSF) at both 780 nm and at 930 nm (12 mW and 49 mW excitation power, respectively).

All imaging was performed at atmospheric pressure, precluding reversible pressure dependent fluorescence intensity changes in GFP itself. Moreover, GFP is extremely pressure stable and does not unfold until above 1,050 MPa (10 kbar) (Ehrmann et al., 2001; Scheyhing et al., 2002). In the present work, pressure shocks were performed at much lower pressure, 60 MPa. Moreover, we have shown previously that there is no irreversible effect of pressure up to 100 MPa on the molecular brightness (= quantum yield or counts per second per molecule) of GFP (Bourges et al., 2020) in live bacterial cells.

### Scanning number and brightness (sN&B) imaging and analyses

sN&B was developed to allow for quantitative analysis of the number of fluorescent molecules in living cells (Digman et al., 2008; Ferguson et al., 2011, 2012). To perform sN&B measurements, a series of very rapid raster scans are obtained (for these experiments, 25 frames were acquired) for each field of view (FOV). A pixel dwell time of 40 μs was used, which is faster than the diffusion time of GFP in cells (~5 μm^2^/s,) (Ferguson et al., 2011) to allow for measurement of the fluorescence fluctuations. The average fluorescence intensity, <*F_GFP_*>, of the diffusing GFP molecules and the variance of their fluorescence, σ^2^, were used to calculate the shot noise corrected molecular brightness of GFP (*e_GFP_*) at each pixel in each bacterial cell according to equation 1.


(1) $$e_{GFP}=\frac{\sigma^{2}}{\langle F_{GFP}\rangle }-1=B-1.$$

Then *e_GFP_* was averaged across all bacterial cells to provide the average molecular brightness of GFP (<*e_GFP_>*). Using the average molecular brightness of GFP, the absolute number of GFP molecules (*n_GFP_*) within the effective volume (*V_eff_*) defined by the point spread function (PSF) of the excitation laser was determined for each pixel in each bacterial cell from the average fluorescence over all scans at that pixel according to equation 2.


(2) $$n_{GFP}=\frac{\langle F_{GFP}\rangle }{\langle e_{GFP}\rangle }\text{.}$$

Values of *n_GFP_* were averaged over all quantified pixels within each cell, <*n_GFP_>*, and correspond to the absolute concentration of GFP (number of GFP molecules in the *V_eff_*) in each cell.

In some cases, GFP expression was so high that it saturated the detectors. In these cases, the excitation intensity was lowered such that the detected fluorescence intensity was sufficiently below the limit of the detector. To accurately compare data acquired with different excitation intensities, the fluorescence intensities were first normalized to the highest excitation intensity according to equation 3.


(3) $$F_{\text{norm}}=F_{i}*{\left(\frac{E_{\text{norm}}}{E_{i}}\right)}^{2}$$

where F_norm_ is the normalized fluorescence intensity, F_i_ is the initial fluorescence intensity, E_norm_ is the normalized excitation intensity, and E_i_ is the initial excitation intensity. Background subtraction and sN&B analyses (see below) were only carried out after fluorescence intensity normalization, as the background fluorescence was always measured with E_norm_.

sN&B analyses were carried out using the Patrack software (Espenel et al., 2008) to manually segment cells for single cell resolution. Prior to calculation of GFP brightness and number, background fluorescence, determined from imaging strain MG1655 or AN62 with no GFP producing plasmids, was subtracted from the fluorescence intensity at each pixel. To avoid artfacts that arise from imaging along the boundaries of cells due to the diffraction-limited PSF, only the central pixels were used to determine the average fluorescence intensity in each cell. The distribution of the <*n_GFP_*> value for each cell from all FOV for a given condition was then plotted and compared between populations of cells that received no shock or a heat or pressure shock. From the averages of the histogram distributions, the percent change in promoter activity after either heat or pressure shock was calculated and averaged for 3 separate experiments for each strain and condition. Pairwise *T* tests were then performed for all promoter fusion strains under all conditions (Supplementary Table S4).

### Protein structure visualization

Protein structure files were taken from the protein databank (PDB). Files were then viewed in pymol (The PyMOL Molecular Graphics System, Version 3.0 Schrödinger, LLC), and key residues were emphasized using visualization tools in the software.

## Results

### Quantification of the transcriptional response to heat shock for heat shock genes

**Figure 2:**
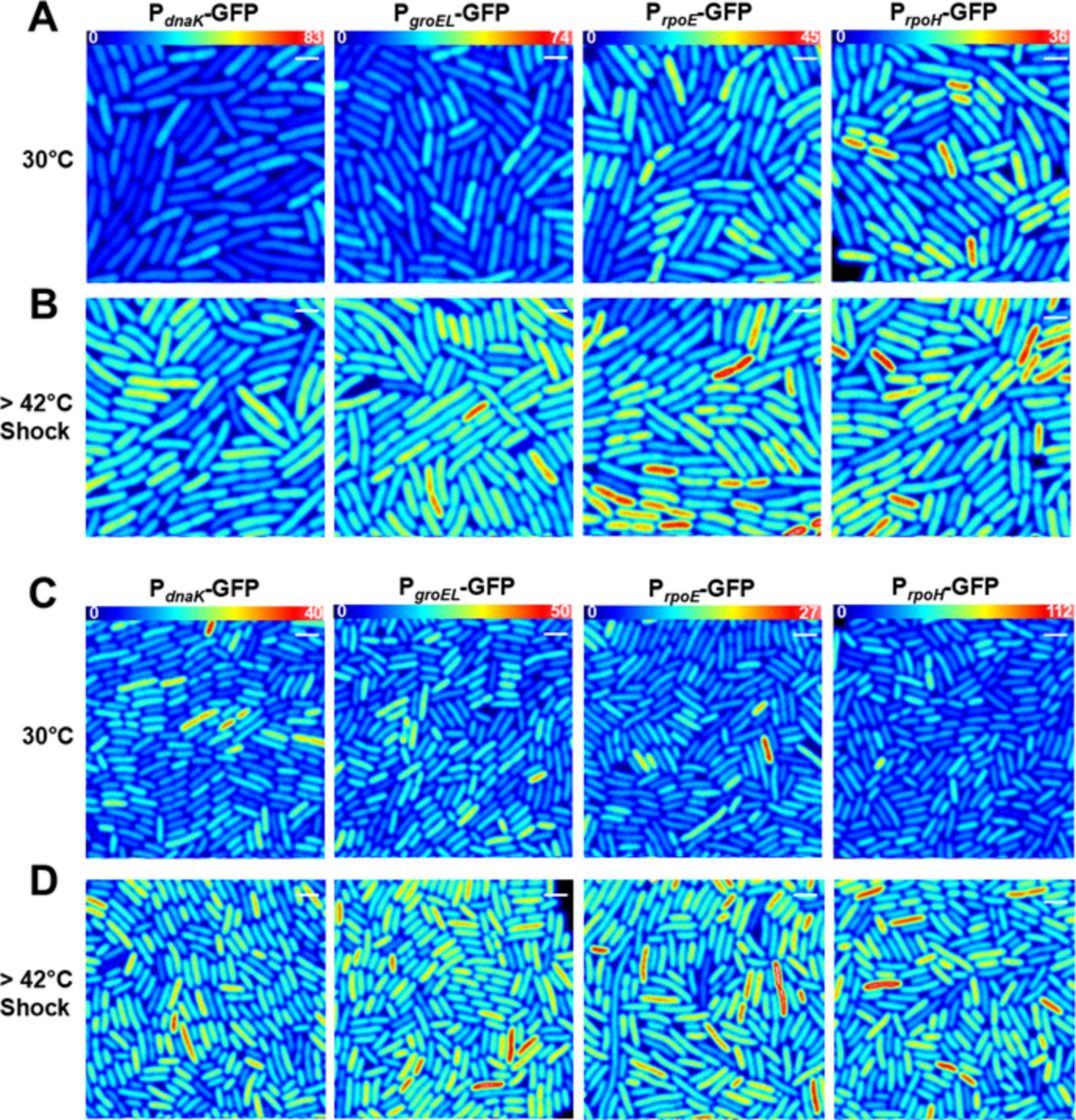
Transcriptional heat shock response in single cells. Results are presented in **(A,B)** *E. coli* MG1655 or **(C,D)** the *E. coli* AN62 strain. Representative fluorescence intensity images for each promoter fusion after growth at 30°C **(A,C)** without any shock and **(B,D)** after a 15-min, 42°C heat shock. Full intensity scales are **(A,B)** MG1655 *P_dnaK_* (0–83), MG1655 *P_groEL_* (0–74), MG1655 *P_rpoE_* (0–45), and MG1655 *P_rpoH_* (0–36). **(C,D)** AN62 *P_dnaK_* (0–40), AN62 *P_groEL_* (0–50), AN62 *P_rpoE_* (0–27), and AN62 *P_rpoH_* (0–112). Spatial scale bars (white) are 2 μm.

To quantify the transcriptional response to heat shock we performed 2-photon sN&B imaging on *E. coli* strains MG1655 and AN62 bearing GFP plasmid-borne promoter fusions of the four major heat shock genes *dnaK, groEL, rpoE, and rpoH*. Because the mRNA and protein produced is the same (GFP), beyond short 5’-UTR regions specific to each promoter, for all promoters in both strains, these experiments monitor directly changes in promoter activity, as opposed to differences in the amount of RNA transcript or HS protein produced. The heat shock transcriptional response was characterized before (at 30°C) and after a 15-min 42°C heat shock similar to previous heat shock studies (Gross et al., 1984; Taylor et al., 1984; Grossman et al., 1984; Erickson et al., 1987). Single cell resolution was achieved via manual cell segmentation as described in the Methods section. Dividing the fluorescence intensity averaged over all central pixels in each cell by the molecular brightness of GFP, *e_GFP_*, calculated using equation 1, yielded the average absolute number of GFP molecules in the *V_eff_* in each cell, <*n_GFP_*> equation 2 which corresponds to the absolute concentration of GFP in each cell. Both MG1655 (Figure 2A) and AN62 (Figure 2C) exhibited basal levels of expression prior to heat shock due to RNA polymerase recruitment via σ^70^ (or σ^32^ in the case of *dnaK*). In some cases, GFP expression was so high that the excitation intensity was decreased to avoid oversaturation of the detector. To ensure comparability between all promoter fusions, fluorescence intensity values were normalized to the highest excitation intensity using equation 3 (see Methods section). Additionally, since these strains bear the promoter GFP fusions on plasmids, the initial expression levels (intensities) for repeat experiments varied, as well as between strains and promoters. Thus, intensities could not be compared either between promoters or strains. Rather, it is the magnitude of the fractional change in expression after shock that is significant and should be compared.

**Figure 3:**
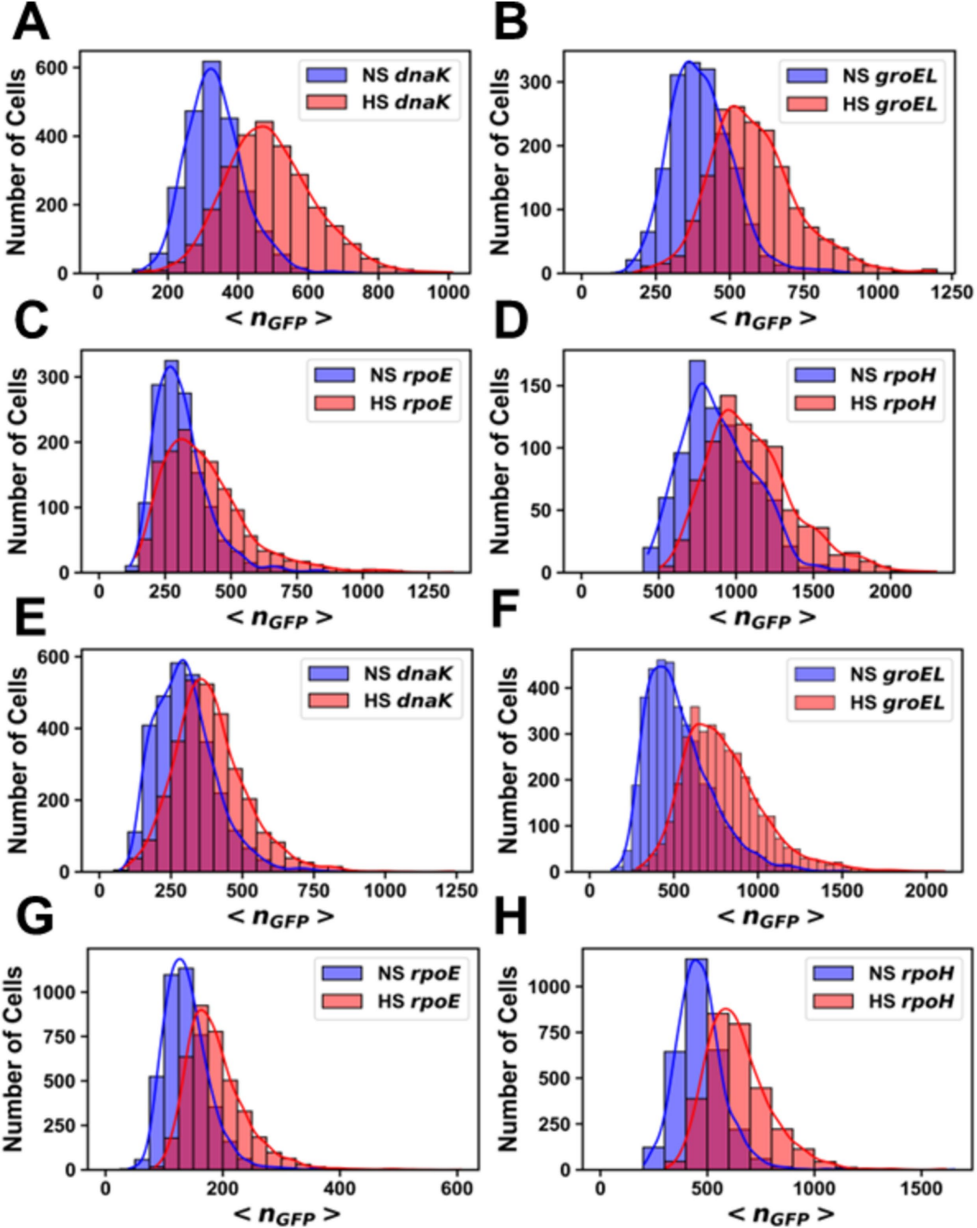
Histograms of the number of molecules of GFP per cell before and after heat shock. Promoter fusions for **(A)** MG1655 *P_dnaK_*, **(B)** MG1655 *P_groEL_*, **(C)** MG1655 *P_rpoE_*, **(D)** MG1655 P*_rpoH_*, **(E)** AN62 *P_dnaK_*, **(F)** AN62 *P_groEL_*, **(G)** AN62 *P_rpoE_* and **(H)** AN62 *P_rpoH_*. Cells that received a heat shock (HS) are colored red, and cells that did not receive a heat shock are colored blue (NS). Cells were grown at 30°C prior to heat shock at 42°C for 15 min. The absolute numbers of GFP molecules were determined by sN&B analysis. Note that data are plotted on different x and y axes for different experiments due to differences in basal levels (plasmid copy number and intrinsic promoter activity). Axes have been optimized to allow comparison of the shock vs. no shock samples.

**Figure 4:**
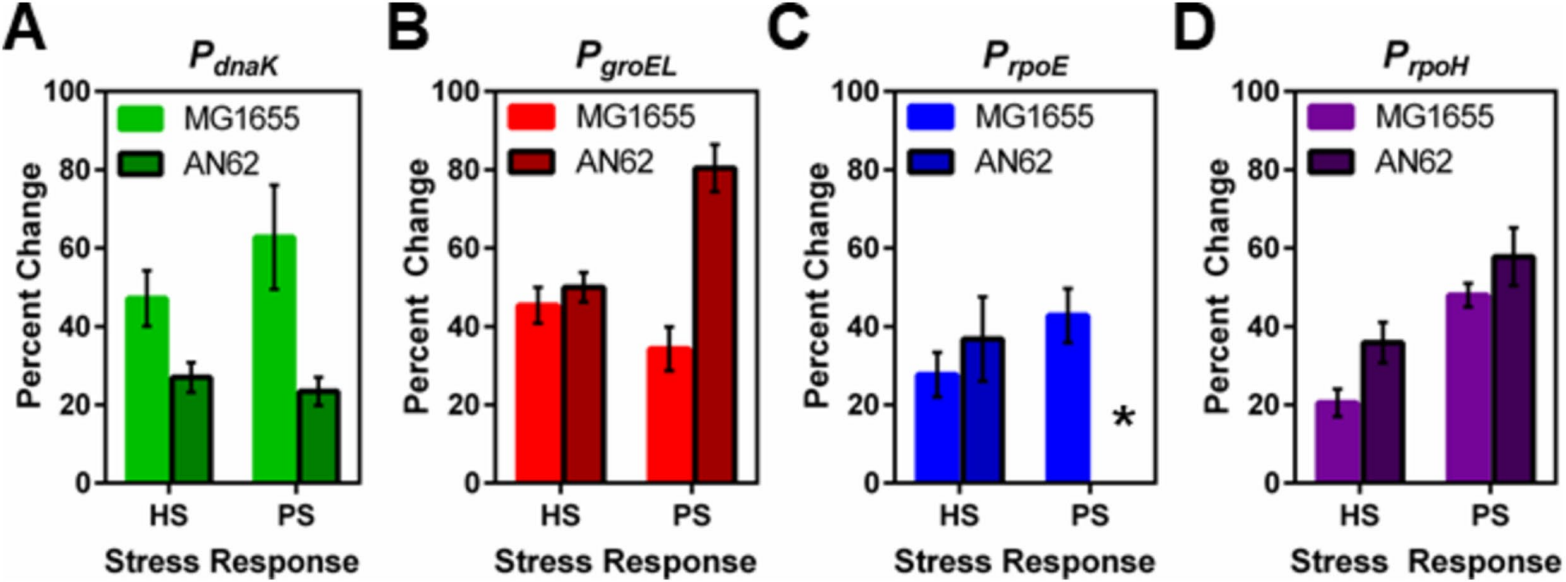
Comparison between the heat and pressure shock responses in *E. coli* MG1655 and AN62. The percent change in the number of molecules of GFP produced after heat and pressure shocks are compared for the promoter fusions for **(A)** *P_dnaK_*, **(B)** *P_groEL_*, **(C)** *P_rpoE_*, and **(D)** *P_rpoH_*. Because of the stochastic response to pressure shock for the *P_rpoE_* promoter in AN62, no percent change was calculated, indicated by the asterisk. Error bars are one standard deviation of the average of three biological replicates.

After heat shock, an increase in promoter activity, as evidenced by an increase in the value of <*n_GFP_ >* for each cell, was observed for all promoter fusion constructs in both the MG1655 (Figure 2B) and AN62 (Figure 2D) strains. Histograms of <*n_GFP_ >* for all promoter fusion constructs in both the MG1655 and AN62 strains showed a clear increase in expression upon heat shock (Figure 3). Only the *P_dnaK_* and *P_rpoE_* promoter fusions exhibited any significant change in the width of the distributions, corresponding to an increase in biological noise after heat shock (Figures 3A,C). Interestingly, the heat shock transcriptional responses of the chaperone promoters, *P_dnaK_* (47%) and *P_groEL_* (45%) were stronger than those of the alternative sigma factor promoters, *P_rpoH_* (21%) and *P_rpoE_* (28%) (*p* values in Supplementary Table S4; Figure 4). The magnitudes of the transcriptional heat shock responses observed here are consistent with previous studies (Erickson et al., 1987; Riehle et al., 2003; Gunasekera et al., 2008; Ying et al., 2013, 2015; Kim et al., 2020). Since the responses are transient, the actual timing of our measurements after heat shock (~8–10 min) could impact the measured magnitude of the response in comparison to prior results. Note also that post-transcriptional (protein level) HS responses have been shown to be much larger than transcriptional HS responses (Lemaux et al., 1978; Herendeen et al., 1979; Erickson et al., 1987). In contrast to the parental strain, in AN62, the heat shock response of *P_dnaK_* (27%) was only about half as large as that of *P_groEL_* (50%) and was also significantly smaller than the responses of both alternative sigma factor promoters*, P_rpoH_* (36%) and *P_rpoE_* (37%) (Figure 4; *p* values in Supplementary Table S4). Comparing AN62 to MG1655, *P_dnaK_* was upregulated much less after heat shock in the pressure-adapted strain, and the promoters for the alternative sigma factors, *P_rpoH_* and *P_rpoE_*, were upregulated significantly more (Supplementary Table S4). Taken together, all promoter fusions in both the MG1655 and AN62 strains exhibited robust, yet distinct, transcriptional heat shock responses.

### Heat shock genes exhibit a transcriptional response to pressure shock

**Figure 5:**
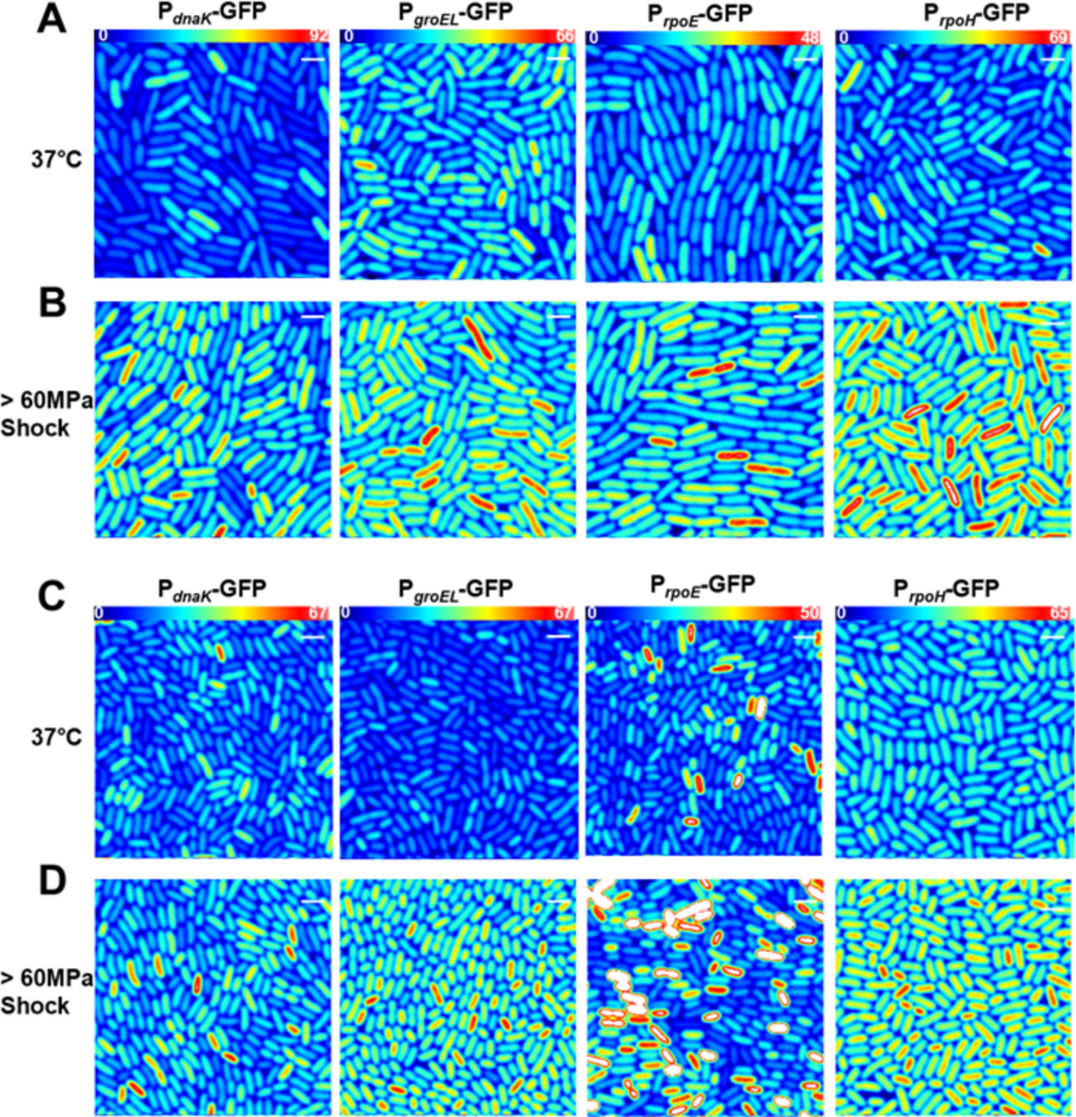
Transcriptional pressure-induced heat shock response in single cells in **(A,B)** *E. coli* MG1655 or **(C,D)** the *E. coli* AN62 strain. Representative fluorescence Intensity images for each promoter fusion after growth at 37°C **(A,C)** without any shock and **(B,D)** after a 15 min, 60 MPa pressure shock. Full intensity scales are **(A,B)** MG1655 *P_dnaK_* (0–92), MG1655 *P_groEL_* (0–66), MG1655 *P_rpoE_* (0–48), and MG1655 *P_rpoH_* (0–69). **(C,D)** AN62 *P_dnaK_* (0–67), AN62 *P_groEL_* (0–67), AN62 *P_rpoE_* (0–50), and AN62 *P_rpoH_* (0–65). Spatial scale bars (white) are 2 μm.

**Figure 6:**
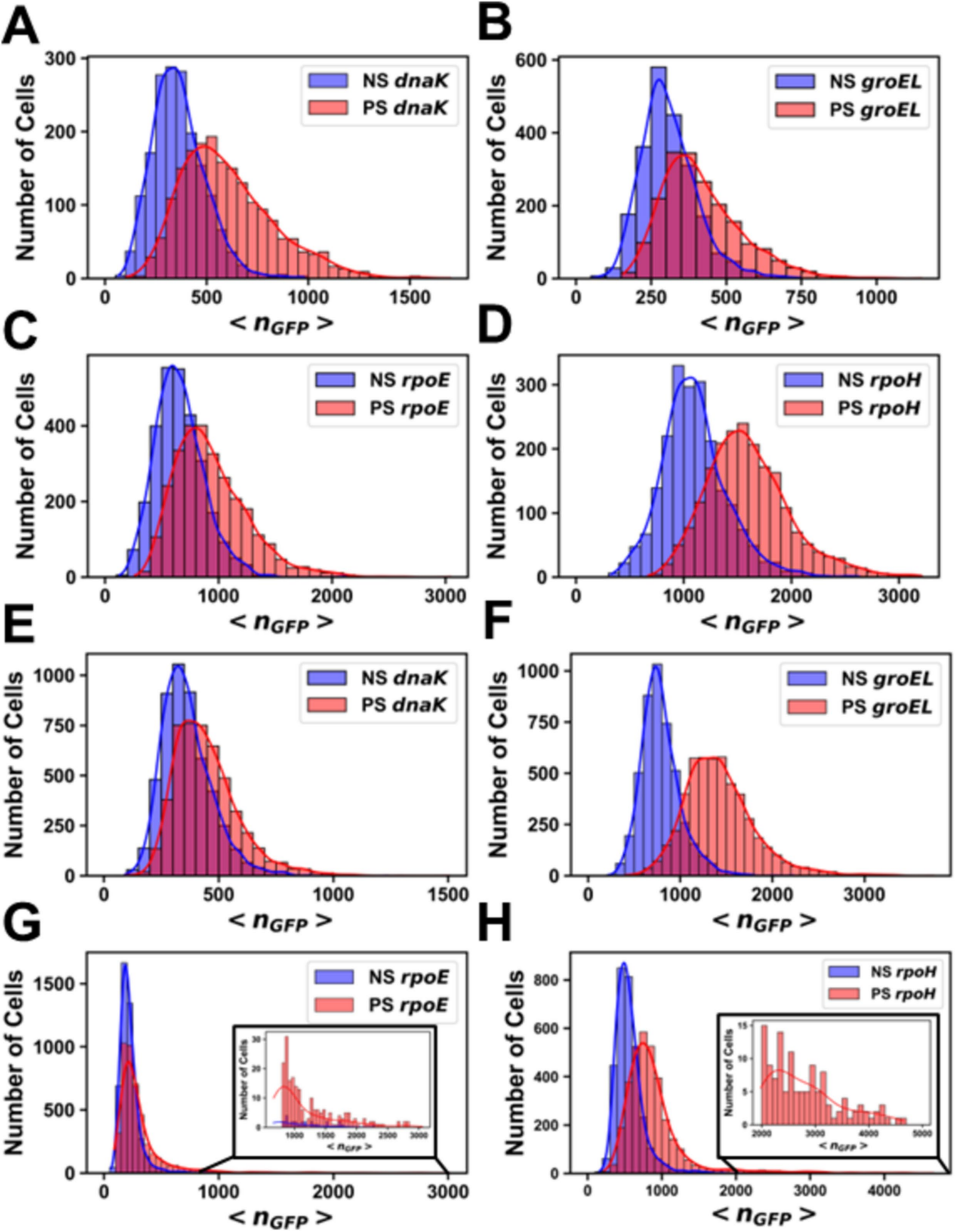
Histograms of the number of molecules of GFP per cell before and after pressure shock. Promoter fusions for **(A)** MG1655 *P_dnaK_*, **(B)** MG1655 *P_groEL_*, **(C)** MG1655 *P_rpoE_*, **(D)** MG1655 *P_rpoH_*, **(E)** AN62 *P_dnaK_*, **(F)** AN62 *P_groEL_*, **(G)** AN62 *P_rpoE_* and **(H)** AN62 *P_rpoH_*. Cells that received a pressure shock (PS) are colored red, and cells that did not receive a pressure shock are colored blue (NS). Cells were grown at 37°C prior to pressure shock at 60 MPa for 15 min. The absolute numbers of GFP molecules were determined by sN&B analysis. Note that different x and y axes are used due to the different total numbers of cells at any given *n_GFP_* value for each experiment and also the different ranges of protein concentrations measured. Axes have been optimized to allow comparison of the shock vs. no shock samples.

It has been reported that *E. coli* mounts a heat shock response after a pressure shock (Welch et al., 1993; Aertsen et al., 2004). To quantify this pressure-induced heat shock response, each promoter fusion strain was subjected to a 15-min 60 MPa pressure shock after growth at 37°C. The magnitude of the pressure shock, 60 MPa, was chosen because it is a sub-lethal pressure shock for MG1655 *E. coli* and is just below the maximum pressure at which the piezotolerant AN62 strain will grow (Marietou et al., 2015). Because AN62 is piezotolerant and not piezophilic, we hypothesized that a 60 MPa pressure shock would still act as a stressor for this strain. Similar to the results above for heat shock, all promoters exhibited basal levels of transcriptional activity (Figures 5A,C) when grown at 0.1 MPa (atmospheric pressure), although as noted above, differences in plasmid copy numbers between strains and within strains for different experiments precludes direct comparison of the basal levels. In general, fluorescence intensity values for basal expression were higher at 37°C compared to 30°C (Figures 3, 6). Due to the especially large amount of basal GFP expression from some promoters, the excitation intensity was lowered to avoid saturation of the detectors and the fluorescence intensity was normalized equation 3. Note that raw intensity values are shown in the images. After pressure shock and return to atmospheric pressure, the absolute concentration of GFP, <*n_GFP_>*, produced from all promoter fusions increased in both MG1655 and AN62, as indicated by the warmer colored cells in the fluorescence intensity heat map images (Figures 5B,D). Note that GFP structure and fluorescence is not affected by 60 MPa pressure *in vitro* (Ehrmann et al., 2001; Scheyhing et al., 2002), and that we have shown previously that GFP fluorescence, itself, is not perturbed by pressure shock *in vivo* (Bourges et al., 2020). Moreover, we confirmed in this study that pressure-induced upregulation was not a general phenomenon, as expression of GFP from the non-heat shock, *P_BAD_*, promoter in presence of arabinose showed no change after pressure shock (Supplementary Figure S1).

Analysis by sN&B yielded the distributions of <*n_GFP_>* per cell before and after pressure shock (Figure 6). In the MG1655 strain after pressure shock, *P_dnaK_* activity increased the most (63%), while the increase for *P_groEL_* (34%), *P_rpoE_* (43%), and *P_rpoH_* (48%) were smaller and similar to each other (*p*-values in Supplementary Table S4; Figure 4). In addition, all the MG1655 promoter fusions exhibited a significant increase in both the mean and the variance of promoter expression distributions after pressure shock (Figures 6A–D). In strain AN62, as observed for the heat shock response, *P_dnaK_* activity increased the least (23%), while *P_groEL_* activity increased the most (80%) (*p* values in Supplementary Table S4; Figure 4). The increased activity of the alternative sigma factor promoter, *P_rpoH_*, (58%) was intermediate. Prior to pressure shock, the cell-to-cell variance in *P_rpoE_* activity in strain AN62 was significant (Figure 5C). Furthermore, *P_rpoE_* and *P_rpoH_* displayed highly stochastic expression patterns after pressure shock, as evidenced by the large tail on the distributions extending far beyond the mean (Figures 5C,D, 6G,H, insets). For strain PrpoE, after pressure shock, many cells exhibited little to no response, while ~30–40% of cells responded very dramatically to pressure, increasing the number of molecules of GFP by up to ~10-fold beyond the mean prior to shock (Figures 6G,H, insets). In particular, because the *P_rpoE_* response was so heterogeneous, the percent change in promoter activity is not particularly informative and for this reason is not provided (Figure 4C). Interestingly, in strain AN62, the pressure-induced heat shock response of *P_groEL_* was larger than its response to temperature, with larger increases in both the mean and the variance of the <*n_GFP_*> distributions (Figures 4, 6E,F; *p* values in Supplementary Table S4). Moreover, the responses to pressure shock of the two chaperone promoters were inversed in strain AN62 compared to strain MG1655 (Figures 4A,B). In AN62, *P_groEL_* showed a larger pressure-induced heat shock response than *P_dnaK_* while in MG1655 *P_dnaK_* experienced a larger increase in promoter activity after pressure shock than *P_groEL_* (*p* values in Supplementary Table S4).

### The heat shock response to pressure is distinct from the response to heat

We were interested to compare the heat-induced heat shock response in both strains to their pressure-induced heat shock responses to probe for any differences in mechanism. For *P_dnaK_*, while we did not observe any statistically significant larger pressure-induced heat shock response compared to the heat-induced response in either strain, MG1655 clearly demonstrated a more robust response from *P_dnaK_* to both heat and pressure shocks than AN62 (Figure 4A; *p* values in Supplementary Table S4). *P_groEL_* in the AN62 strain showed a much stronger response to pressure shock than to heat shock, while in MG1655, there was a slightly stronger response to heat shock than pressure shock (Figure 4B; *p* values in Supplementary Table S4). Only in strain MG1655 did *P_rpoE_* exhibit a general upregulation response to pressure, although this promoter responded to heat shock in both MG1655 and AN62 (Figure 4C). In contrast, in strain AN62 the response to pressure of *P_rpoE_* was highly stochastic (Figure 6G). Of all the promoter fusions studied, only the promoter for the main heat shock sigma factor, *P_rpoH_*, showed a larger response to pressure shock than to heat shock in both strains (Figure 4; *p* values in Supplementary Table S4).

## Discussion

### Both *Escherichia coli* MG1655 and pressure-adapted AN62 exhibit a pressure-induced transcriptional heat shock response

It has been shown previously that in *E. coli* strain MG1655 there is an increase in DnaK and GroEL protein levels during pressure shock (Welch et al., 1993). A rather long-term transcriptional heat shock response to pressure shock in this strain has been reported for *dnaK, lon, and clpPX* (Aertsen et al., 2004). We have confirmed and quantified a transcriptional pressure-induced heat shock response for several key heat shock promoters, *P_dnaK_, P_groEL_*, *P_rpoH_*, and *P_rpoE_* in both MG1655, as well as for strain AN62, adapted in the laboratory to grow at high pressure (Marietou et al., 2015). We note that the single cell resolution and timescale of our observations (performed <10 min after the shock) is distinct from previous studies. It is important to note, as well, that in our studies, the observed upregulation of promoter activity is not due to a change in mRNA stability [as was the case for transcription from the *P_rpoH_* during heat shock (Morita et al., 1999)], since our readout for the activity of all promoters in all conditions is the number of GFP molecules produced (i.e., the same GFP mRNA, differing only in the 5’UTR for each promoter).

### The transcriptional response to pressure shock is unique and adaptable

The transcriptional pressure-induced heat shock response is distinct from the heat shock response. For strain MG1655, pressure shock elicited an equivalent (*P_dnaK_*) or stronger transcriptional upregulation than heat shock for all promoters. In strain AN62, the transcriptional pressure shock dependent heat shock response was complex. It was found to be more robust for *P_groEL_* and *P_rpoH_* than heat shock in either strain, while the response to either heat or pressure shock for *P_dnaK_* was the smallest. Interestingly, *P_rpoE_* and to a lesser extent, *P_rpoH_*, responded stochastically to pressure shock in strain AN62. It is well established that higher pressures disfavor protein aggregation (disaggregation being the main function of DnaK), while favoring protein unfolding (refolding being the main function of GroEL). It is conceivable that, whatever the underlying mechanism, increased GroEL production in strain AN62 could confer some advantage for growth at high pressure.

We wondered what might be the molecular basis for these distinct transcriptional responses to pressure shock in AN62 relative to the parent strain? The most direct mechanisms would implicate transcription, itself, with any differences between promoters arising from differential transcription of their 5’UTR regions, since the coding region corresponds in all cases to GFP (Supplementary Table S2). Strain AN62 harbors only 12 mutations in coding regions of its genome, in addition to five intergenic mutations, three of which are near the gene for tRNA-Gly (Supplementary Table S1; Allemann et al., 2024). Of the mutations in coding sequences, only three affect proteins directly implicated in transcription. The others involve transporters and metabolic enzymes. Of those mutations in genes coding for proteins implicated in transcription, one is a transcriptional activator for the cysteine regulon, which is not involved in the HS response. Another is found in the *rho* terminator gene. However, *rho* mutations are unlikely to be implicated in differential HS promoter activity since no *rho* termination sites are present in the 5’UTR regions of the HS GFP promoter fusions (Supplementary Table S2; Naville et al., 2011).

**Figure 7:**
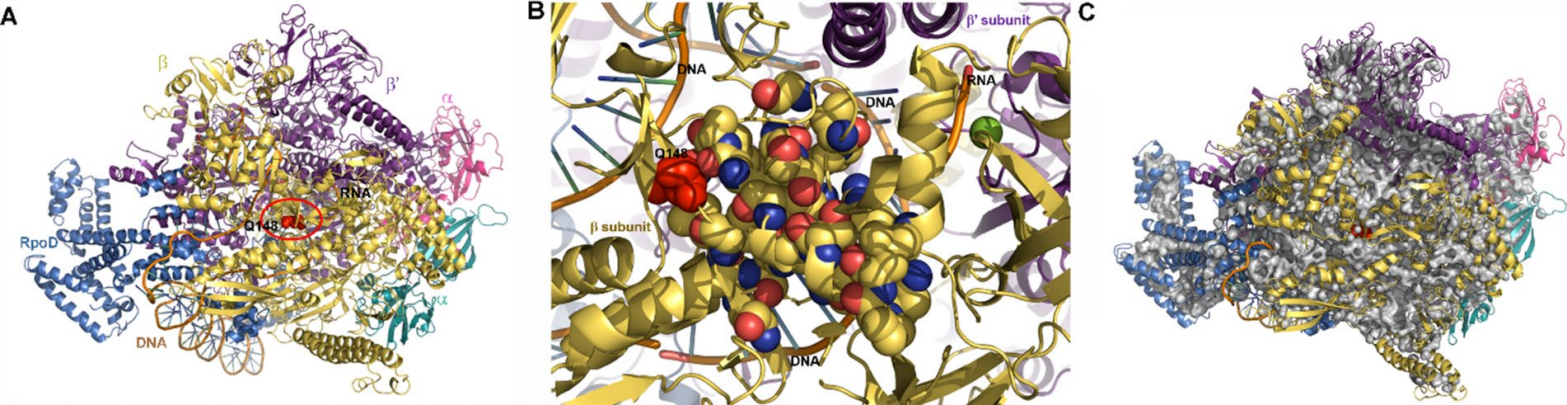
Visualization of RNA polymerase initiation complex. **(A)** The structure of *E. coli* RNA polymerase transcription initiation complex (Zuo and Steitz, 2015) visualized using Pymol (see methods section). Note that residue in the *β* subunit (yellow) of WT RNAP (BQ148) that is mutated to H in AN62 is shown in red spheres and inside a red circle. The *α*, *β* and *β*’ subunits are labeled according to their color. The *ω* subunit is at the back and not visible in this view. The transcribed and non-transcribed DNA, as well as the nascent RNA are also labeled. **(B)** Zoomed in image of the cluster of mutations in the RpoB subunit of *E. coli* RNAP that confer rifampicin resistance. Note that the 21 mutations conferring resistance to rifampicin (yellow CPK spheres), including Q148 in WT RNAP (red spheres and also labeled) are found in the vicinity of the transcription bubble and the mRNA transcript. DNA and RNA are shown in orange ribbon, while bases are shown as blue-green sticks. **(C)** Internal cavities in RNAP. Cavities were calculated using Pymol with a detection radius of 4 solvent molecules and a detection cutoff of 3 solvent molecules. Cavities are shown in gray and Q148 in red spheres. **(A,C)** The *α* subunit of RpoB is colored in magenta, the α’ subunit in aqua, the *β* subunit is colored yellow, the *β*’ subunit in violet and the RpoD subunit in blue.

In contrast, the mutation in *rpoB* which leads to an amino acid substitution (glutamine to histidine) at position 148 in the *β*-subunit of RNA polymerase (RNAP) could conceivably contribute to the observed differential responses of the two strains to pressure shock. The Q148➔H mutation is very close to the transcription bubble and the nascent mRNA, as shown in the structure of the *E. coli* RNAP initiation complex (Figures 7A,B; Zuo and Steitz, 2015). The large number of internal cavities in the RNAP structure (Figure 7C), particularly between the open complex bubble and the mutation, could render this region, and thus RNAP activity, pressure-sensitive, affecting differentially the WT and AN62 enzymes.

While additional stress response mechanisms could certainly contribute to the distinct pressure-induced heat shock responses in strain AN62, the hypothesis that the Q148H mutation in *rpoB* might contribute to this phenomenon is supported by the fact that this substitution is one of over 20 single site mutations located within the rifampicin binding site of RNAP known to confer rifampicin resistance to *E. coli* (Jin et al., 1988; Jun and Gross, 1988; Goldstein, 2014; Molodtsov et al., 2017). The resistance conferring (Rif^r^) mutations, in addition to altering the affinity for rifampicin, lead to significant changes in transcriptional initiation, pausing, elongation and termination efficiency in absence of drug, and have been used to elucidate RNAP functional mechanisms (Jun and Gross, 1988; Jun and Gross, 1989; Landick et al., 1990; Molodtsov et al., 2017; Meenakshi and Hussain Munavar, 2018). Rif^r^ mutations in the β subunit of RNAP have been shown to have pleiotropic effects, as well. They lead to slow growth (Jun and Gross, 1989; Reynolds, 2000), which is known to be strongly dependent upon transcriptional capacity (Izard et al., 2015; Zhang et al., 2020). Indeed, the growth rate of AN62 is slower than that of MG1655 (Marietou et al., 2015). Moreover, Rif^r^ mutations in the RNAP *β* subunit have been shown to result in both upregulation and down-regulation of hundreds of genes (Meenakshi and Hussain Munavar, 2018). Interestingly, Rif^r^ mutations (including one, R143L, quite close to the Q148H substitution in AN62) were selected in absence of rifampicin in a laboratory evolution experiment that involved adaptation to growth at high temperature (Rodríguez-Verdugo et al., 2013).

In contrast to similar sizes for AN62 and the parental MG1655 strains reported previously (Marietou et al., 2015), we have observed consistently that the cells in strain AN62 are significantly smaller (50%) when grown at atmospheric pressure. The discrepancy may stem from the fact that cells were fixed before imaging in the previous study. While the mechanism underlying the difference in size is outside the scope of the current study, we offer one possible hypothesis. Cell division in *E. coli* is licensed by DNA replication, but size is controlled by a division adder, i.e., sufficient accumulation (relative to growth rate) of initiators and precursors required for cell division and maintenance of their production proportional to volume growth (Si et al., 2019). Thus, the smaller size in strain AN62 could result from differential scaling between growth (which, as noted above, is slower than the parental strain) and the rate of production of proteins required for division (e.g., FtsZ). Interestingly, *ftsZ* and *ftsA* (which recruits FtsZ to the septum) are among the genes shown to be upregulated by certain Rif^r^ mutations, while the gene for a repressor of division, *sulA*, was found to be the most strongly downregulated (Meenakshi and Hussain Munavar, 2018). Future work will be aimed at testing the role of the *rpoB* mutation in supporting growth of strain AN62 at high pressure.

### RpoE may act as a pressure sensor for the pressure-induced heat shock response

As noted above, upregulation of *P_rpoE_* in AN62 after pressure shock was limited and strongly stochastic compared to MG1655, where it is upregulated robustly. While more work is needed to understand this differential expression pattern for the two strains, we hypothesize the difference may at least partially arise from differences in membrane composition of the two strains. Under homeostatic conditions, RpoE is sequestered at the membrane by the integral membrane protein RseA and is only released upon stress to the membrane and/or extra cytoplasmic/membrane proteins (Peñas et al., 1997; Missiakas et al., 1997; Klein et al., 2016). Membranes are very susceptible to pressure changes (e.g., Lakowicz and Thompson, 1983; Winter and Jeworrek, 2009; Winnikoff et al., 2024), with significant decreases in fluidity resulting from increased pressure. We hypothesize that the pressure-induced decrease in membrane fluidity, could lead to release of RpoE, which would then upregulate *rpoH* and its own expression. Since the membranes of the AN62 strain contain a larger fraction of unsaturated fatty acids than the MG1655 strain (20.02% 18:1 ω7c vs. 9.5%) (Marietou et al., 2015), the membrane of AN62 may experience less membrane stress due to pressure shock, resulting in the observed limited *rpoE* upregulation in the pressure adapted strain. The very strong expression in the small fraction of AN62 cells that do respond to pressure shock could arise from differences in RNAP function at high pressure in the pressure-adapted strain.

## Concluding remarks

The present results both confirm and quantify a pressure-induced transcriptional heat shock response in *E. coli*. This response to pressure shock, is distinct from the heat shock response and distinct between the parent and pressure-adapted strain for several promoters. Our results suggest that a rifampicin resistance mutation in the *β* subunit of RNAP in the pressure-adapted strain could contribute to the differential responses. Another intriguing hypothesis that stems from our observations is that RpoE and its anti-sigma factors may act as a membrane-linked pressure sensors to aid in activating the pressure-induced heat shock response in the parent strain, while the different membrane composition in AN62 could protect the pressure-adapted strain. Taken together, our results point to the importance of transcription and membrane stability in pressure adaptation and provide a foundation for future studies aimed at understanding organismal adaptation to, and even preference for, high pressure.

## Data Availability

The original contributions presented in the study are publicly available. This data can be found here: 10.6084/m9.figshare.27310554.
