# Supplementary material for: Response and adaptation of the transcriptional heat shock response to pressure
Source: Front Microbiol. 2024 Nov 18;15:1470617. doi: 10.3389/fmicb.2024.1470617 (PMC11609203; doi:10.3389/fmicb.2024.1470617)
Supplement: Supplementary file 1 [file Data_Sheet_1.pdf]

Supplemental Materials
for

Response and Adaptation of the Transcriptional Heat Shock Response to Pressure

Carleton H. Coffin[1,*], Luke A. Fisher[2,*], Sara Crippen[3], Phoebe Demers[3], Douglas H. Bartlett[2,‡] and Catherine A. Royer[3‡]

[1]Graduate Program in Biochemistry and Biophysics, Rensselaer Polytechnic Institute, Troy, NY 12180

[2]Marine Biology Research Division, Scripps Institution of Oceanography, University of California, San Diego, La Jolla, CA 92093-0202

[3]Department of Biological Sciences, Rensselaer Polytechnic Institute, Troy NY, 12180

Table S1 – mutations unique to AN62

Note that several of these mutations are missing in the table published in Allemann et al. (2024) on BioRxiv (https://www.biorxiv.org/content/10.1101/2024.09.24.613341v1). The present list is complete.

position	mutation	annotation	gene	synonyms	description
1098909	G→T	V11F (GTT→TTT)	*ycdX* →		alkaline phosphatase
1151745	T→G	V44G (GTA→GGA)	*acpP* →		acyl carrier protein (ACP)
4513933	G→A	L126F (CTT→TTT)	*fecB* ←		iron-dicitrate transporter subunit
2419024	A→C	I51M (ATT→ATG)	*yfcD* ←		putative NUDIX hydrolase
2898780	C→A	L213F (TTG→TTT)	*ygcU* ←		putative FAD-containing dehydrogenase
2966801	G→T	A545D (GCT→GAT)	*ptsP* ←		fused PTS enzyme: PEP-protein phosphor-transferase (enzyme I)/GAF domain containing protein
3966884	T→G	D156E (GAT→GAG)	*rho* →		transcription termination factor
4057215	A→G	S274P (TCT→CCT)	*glnA* ←		glutamine synthetase
4182574	G→T	D444Y (GAT→TAT)	*rpoB* →		RNA polymerase, beta subunit
4548893	Δ36 bp	coding (86-121/903 nt)	*fimH* →		minor component of type 1 fimbriae
1334738	IS*1* (–) +9 bp	coding (884-892/975 nt)	*cysB* →		N-acetylserine-responsive cysteine regulon transcriptional activator; autorepressor
1457145	IS*4* (-) +11 bp	coding (716-726/1071 nt)	*paaE* →		ring 1,2-phenylacetyl-CoA epoxidase, NAD(P)H oxidoreductase component
1979486	IS*5* (+) +4 bp	intergenic (-271/-264)	*insA* ← / → *uspC*	*uspC (yecG)*	IS1 repressor TnpA/universal stress protein
4392557	+A	intergenic (+10/-26)	*glyX* → / → *glyY*		tRNA-Gly/tRNA-Gly
4392564	T→C	intergenic (+17/-19)	*glyX* → / → *glyY*		tRNA-Gly/tRNA-Gly
4392567	G→A	intergenic (+20/-16)	*glyX* → / → *glyY*		tRNA-Gly/tRNA-Gly
1260896	C→A	intergenic (-93/+32)	*dauA* ← / ← *prs*	*ychM (dauA) prs (dnaR prsA)*	C4-dicarboxylic acid transporter/phosphoribosylpyrophosphate synthase

Table S2: Sequencing results for plasmid GFP promoter fusions. Sequences show the promoter fused transcriptionally to GFPmut2. Underlined are the 5' untranslated regions (UTRs). Green text is the sequence of GFPmut2. The bolded first letter is the transcriptional start site.

Gene	Sequenced GFP promoter Fusion
dnaK P1	**A**ACCGCAGTGAGTGAGTCTGCAAAAAAATGAAAT<br>TGGGCAGTTGAAACCAGACGTTTCGCCCCTATTA<br>CAGACTCACAACCACATGATGACCGAATATATAGT<br>GGAGACGTTTAGATGGGTAAAATAATTGGTATCG<br>ACCTGGGTACTACCAACTCTTGTGTAGCGATTAT<br>GGATGGCACCGGATCCTCTAGATTTAAGAAGGA<br>GATATACATATGAGTAAAGGAGAAGAACTTTTCAC<br>TGGAGTTGTCCCAATTCTTGTTGAATTAGATGGT<br>GATGTTAATGGGCACAAATTTTCTGTCAGTGGAG<br>AGGGTGAAGGTGATGCAACATACGGAAAACTTAC<br>CCTTAAATTTATTTGCACTACTGGAAAACTACCTG<br>TTCCATGGCCAACACTTGTCACTACTTTCGCGTA<br>TGGTCTTCAATGCTTTGCGAGATACCCAGATCAT<br>ATGAAACAGCATGACTTTTTCAAGAGTGCCATGC<br>CCGAAGGTTATGTACAGGAAAGAACTATATTTTTC<br>AAAGATGACGGGAACTACAAGACACGTGCTGAA<br>GTCAAGTTTGAAGGTGATACCCTTGTTAATAGAAT<br>CGAGTTAAAAGGTATTGATTTTAAAGAAGATGGAA<br>ACATTCTTGGACACAAATTGGAATACAACTATAAC<br>TCACACAATGTATACATCATGGCAGACAAACAAAA<br>GAATGGAATCAAAGTTAACTTCAAAATTAGACACA<br>ACATTGAAGATGGAAGCGTTCAACTAGCAGACCA<br>TTATCAACAAAATACTCCAATTGGCGATGGCCCT<br>GTCCTTTTACCAGACAACCATTACCTGTCCACAC<br>AATCTGCCCTTTCGAAAGATCCCAACGAAAAGAG<br>AGACCACATGGTCCTTCTTGAGTTTGTAACAGCT<br>GCTGGGATTACACATGGCATGGATGAACTATACA<br>AA
dnaK P2	**A**CAACCACATGATGACCGAATATATAGTGGAGAC<br>GTTTAGATGGGTAAAATAATTGGTATCGACCTGG<br>GTACTACCAACTCTTGTGTAGCGATTATGGATGG<br>CACCGGATCCTCTAGATTTAAGAAGGAGATATAC<br>ATATGAGTAAAGGAGAAGAACTTTTCACTGGAGT<br>TGTCCCAATTCTTGTTGAATTAGATGGTGATGTTA<br>ATGGGCACAAATTTTCTGTCAGTGGAGAGGGTG<br>AAGGTGATGCAACATACGGAAAACTTACCCTTAA

	ATTTATTTGCACTACTGGAAAACTACCTGTTCCAT GGCCAACACTTGTCACTACTTTCGCGTATGGTCT TCAATGCTTTGCGAGATACCCAGATCATATGAAAC AGCATGACTTTTTCAAGAGTGCCATGCCCGAAG GTTATGTACAGGAAAGAACTATATTTTTCAAAGAT GACGGGAACTACAAGACACGTGCTGAAGTCAAG TTTGAAGGTGATACCCTTGTTAATAGAATCGAGTT AAAAGGTATTGATTTTAAAGAAGATGGAAACATTC TTGGACACAAATTGGAATACAACTATAACTCACAC AATGTATACATCATGGCAGACAAACAAAAGAATG GAATCAAAGTTAACTTCAAAATTAGACACAACATT GAAGATGGAAGCGTTCAACTAGCAGACCATTATC AACAAAATACTCCAATTGGCGATGGCCCTGTCCT TTTACCAGACAACCATTACCTGTCCACACAATCT GCCCTTTCGAAAGATCCCAACGAAAAGAGAGAC CACATGGTCCTTCTTGAGTTTGTAACAGCTGCTG GGATTACACATGGCATGGATGAACTATACAAA
dnaK P3	**A**TATAGTGGAGACGTTTAGATGGGTAAAATAATTG GTATCGACCTGGGTACTACCAACTCTTGTGTAGC GATTATGGATGGCACCGGATCCTCTAGATTTAAG AAGGAGATATACATATGAGTAAAGGAGAAGAACT TTTCACTGGAGTTGTCCCAATTCTTGTTGAATTAG ATGGTGATGTTAATGGGCACAAATTTTCTGTCAG TGGAGAGGGTGAAGGTGATGCAACATACGGAAA ACTTACCCTTAAATTTATTTGCACTACTGGAAAAC TACCTGTTCCATGGCCAACACTTGTCACTACTTT CGCGTATGGTCTTCAATGCTTTGCGAGATACCCA GATCATATGAAACAGCATGACTTTTTCAAGAGTG CCATGCCCGAAGGTTATGTACAGGAAAGAACTAT ATTTTTCAAAGATGACGGGAACTACAAGACACGT GCTGAAGTCAAGTTTGAAGGTGATACCCTTGTTA ATAGAATCGAGTTAAAAGGTATTGATTTTAAAGAA GATGGAAACATTCTTGGACACAAATTGGAATACA ACTATAACTCACACAATGTATACATCATGGCAGAC AAACAAAAGAATGGAATCAAAGTTAACTTCAAAAT TAGACACAACATTGAAGATGGAAGCGTTCAACTA GCAGACCATTATCAACAAAATACTCCAATTGGCG ATGGCCCTGTCCTTTTACCAGACAACCATTACCT GTCCACACAATCTGCCCTTTCGAAAGATCCCAAC GAAAAGAGAGACCACATGGTCCTTCTTGAGTTTG TAACAGCTGCTGGGATTACACATGGCATGGATGA ACTATACAAA

groEL P1 and P2 (σ70 and σ32)	**A**CCAGCCGGGAAACCACGTAAGCTCCGGCGTC<br>ACCCATAACAGATACGGACTTTCTCAAAGGAGAG<br>TTATCAATGAATATTCGTCCATTGCATGATCGCGT<br>GATCGTCAAGCGTAAAGAAGTTGAAACTAAATCT<br>GCTGGCGGCATGGATCCTCTAGATTTAAGAAGGA<br>GATATACATATGAGTAAAGGAGAAGAACTTTTCAC<br>TGGAGTTGTCCCAATTCTTGTTGAATTAGATGGT<br>GATGTTAATGGGCACAAATTTTCTGTCAGTGGAG<br>AGGGTGAAGGTGATGCAACATACGGAAAACTTAC<br>CCTTAAATTTATTTGCACTACTGGAAAACTACCTG<br>TTCCATGGCCAACACTTGTCACTACTTTCGCGTA<br>TGGTCTTCAATGCTTTGCGAGATACCCAGATCAT<br>ATGAAACAGCATGACTTTTTCAAGAGTGCCATGC<br>CCGAAGGTTATGTACAGGAAAGAACTATATTTTTC<br>AAAGATGACGGGAACTACAAGACACGTGCTGAA<br>GTCAAGTTTGAAGGTGATACCCTTGTTAATAGAAT<br>CGAGTTAAAAGGTATTGATTTTAAAGAAGATGGAA<br>ACATTCTTGGACACAAATTGGAATACAACTATAAC<br>TCACACAATGTATACATCATGGCAGACAAACAAAA<br>GAATGGAATCAAAGTTAACTTCAAAATTAGACACA<br>ACATTGAAGATGGAAGCGTTCAACTAGCAGACCA<br>TTATCAACAAAATACTCCAATTGGCGATGGCCCT<br>GTCCTTTTACCAGACAACCATTACCTGTCCACAC<br>AATCTGCCCTTTCGAAAGATCCCAACGAAAAGAG<br>AGACCACATGGTCCTTCTTGAGTTTGTAACAGCT<br>GCTGGGATTACACATGGCATGGATGAACTATACA<br>AA
rpoE P1 and P2 (σ70 and σ24)	**C**TTGCTCAAATTGCAGCTAATGGAGTGGCGTTTC<br>GATAGCGCGTGGAAATTTGGTTTGGGGAGACTTT<br>ACCTCGGATGAGCGAGCAGTTAACGGACCAGGT<br>CCTGGTTGAACGGGTCCAGAAGGGAGATCAGAA<br>AGCCTTTAACTTACTGGTAGTGCGCTATCAGCATA<br>AAGTGGCCTCGAGAGATCCTCTAGATTTAAGAAG<br>GAGATATACATATGAGTAAAGGAGAAGAACTTTTC<br>ACTGGAGTTGTCCCAATTCTTGTTGAATTAGATG<br>GTGATGTTAATGGGCACAAATTTTCTGTCAGTGG<br>AGAGGGTGAAGGTGATGCAACATACGGAAAACT<br>TACCCTTAAATTTATTTGCACTACTGGAAAACTAC<br>CTGTTCCATGGCCAACACTTGTCACTACTTTCGC<br>GTATGGTCTTCAATGCTTTGCGAGATACCCAGAT<br>CATATGAAACAGCATGACTTTTTCAAGAGTGCCA<br>TGCCCGAAGGTTATGTACAGGAAAGAACTATATTT

	TTCAAAGATGACGGGAACTACAAGACACGTGCT<br>GAAGTCAAGTTTGAAGGTGATACCCTTGTTAATA<br>GAATCGAGTTAAAAGGTATTGATTTTAAAGAAGAT<br>GGAAACATTCTTGGACACAAATTGGAATACAACT<br>ATAACTCACACAATGTATACATCATGGCAGACAAA<br>CAAAAGAATGGAATCAAAGTTAACTTCAAAATTAG<br>ACACAACATTGAAGATGGAAGCGTTCAACTAGCA<br>GACCATTATCAACAAAATACTCCAATTGGCGATG<br>GCCCTGTCCTTTTACCAGACAACCATTACCTGTC<br>CACACAATCTGCCCTTTCGAAAGATCCCAACGAA<br>AAGAGAGACCACATGGTCCTTCTTGAGTTTGTAA<br>CAGCTGCTGGGATTACACATGGCATGGATGAACT<br>ATACAAA
rpoE P1B (σ70)	**G**TTTGGTCAGCATAGCATCATGTTGTGCGGATAA<br>ACACCTGCTATTTTAATATTTGTTACAGTTGCTAAA<br>CACGCTGACGCAGGGCGGCGAGAAAAAGAGAA<br>GTTACTGGCTGGTGGAGGATTAGGTGGTGAAATA<br>AAAAGGCCGTTGGGTTACTCTTCAGGCAGTTAAA<br>TGGGCATTTCTACACAGATAATGCGATGTTCAGAT<br>TCTGTAGACTTATAATGATAGATAATGATCCGTCTA<br>CAGCATGACAAACAAAAACAGATGCGTTACGGAA<br>CTTTACAAAAACGAGACACTCTAACCCTTTGCTT<br>GCTCAAATTGCAGCTAATGGAGTGGCGTTTCGAT<br>AGCGCGTGGAAATTTGGTTTGGGGAGACTTTAC<br>CTCGGATGAGCGAGCAGTTAACGGACCAGGTCC<br>TGGTTGAACGGGTCCAGAAGGGAGATCAGAAAG<br>CCTTTAACTTACTGGTAGTGCGCTATCAGCATAAA<br>GTGGCCTCGAGAGATCCTCTAGATTTAAGAAGGA<br>GATATACATATGAGTAAAGGAGAAGAACTTTTCAC<br>TGGAGTTGTCCCAATTCTTGTTGAATTAGATGGT<br>GATGTTAATGGGCACAAATTTTCTGTCAGTGGAG<br>AGGGTGAAGGTGATGCAACATACGGAAAACTTAC<br>CCTTAAATTTATTTGCACTACTGGAAAACTACCTG<br>TTCCATGGCCAACACTTGTCACTACTTTCGCGTA<br>TGGTCTTCAATGCTTTGCGAGATACCCAGATCAT<br>ATGAAACAGCATGACTTTTTCAAGAGTGCCATGC<br>CCGAAGGTTATGTACAGGAAAGAACTATATTTTTC<br>AAAGATGACGGGAACTACAAGACACGTGCTGAA<br>GTCAAGTTTGAAGGTGATACCCTTGTTAATAGAAT<br>CGAGTTAAAAGGTATTGATTTTAAAGAAGATGGAA<br>ACATTCTTGGACACAAATTGGAATACAACTATAAC<br>TCACACAATGTATACATCATGGCAGACAAACAAAA

	GAATGGAATCAAAGTTAACTTCAAAATTAGACACA<br>ACATTGAAGATGGAAGCGTTCAACTAGCAGACCA<br>TTATCAACAAAATACTCCAATTGGCGATGGCCCT<br>GTCCTTTTACCAGACAACCATTACCTGTCCACAC<br>AATCTGCCCTTTCGAAAGATCCCAACGAAAAGAG<br>AGACCACATGGTCCTTCTTGAGTTTGTAACAGCT<br>GCTGGGATTACACATGGCATGGATGAACTATACA<br>AA
rpoE P5 (σ70)	**A**<u>ATGATCCGTCTACAGCATGACAAACAAAAACAG</u><br><u>ATGCGTTACGGAACTTTACAAAAACGAGACACTC</u><br><u>TAACCCTTTGCTTGCTCAAATTGCAGCTAATGGA</u><br><u>GTGGCGTTTCGATAGCGCGTGGAAATTTGGTTTG</u><br><u>GGGAGACTTTACCTCGGATGAGCGAGCAGTTAA</u><br><u>CGGACCAGGTCCTGGTTGAACGGGTCCAGAAG</u><br><u>GGAGATCAGAAAGCCTTTAACTTACTGGTAGTGC</u><br><u>GCTATCAGCATAAAGTGGCCTCGAGAGATCCTCT</u><br><u>AGATTTAAGAAGGAGATATACAT</u>ATGAGTAAAGGA<br>GAAGAACTTTTCACTGGAGTTGTCCCAATTCTTG<br>TTGAATTAGATGGTGATGTTAATGGGCACAAATTT<br>TCTGTCAGTGGAGAGGGTGAAGGTGATGCAACA<br>TACGGAAAACTTACCCTTAAATTTATTTGCACTAC<br>TGGAAAACTACCTGTTCCATGGCCAACACTTGTC<br>ACTACTTTCGCGTATGGTCTTCAATGCTTTGCGA<br>GATACCCAGATCATATGAAACAGCATGACTTTTTC<br>AAGAGTGCCATGCCCGAAGGTTATGTACAGGAA<br>AGAACTATATTTTTCAAAGATGACGGGAACTACAA<br>GACACGTGCTGAAGTCAAGTTTGAAGGTGATAC<br>CCTTGTTAATAGAATCGAGTTAAAAGGTATTGATT<br>TTAAAGAAGATGGAAACATTCTTGGACACAAATT<br>GGAATACAACTATAACTCACACAATGTATACATCA<br>TGGCAGACAAACAAAAGAATGGAATCAAAGTTAA<br>CTTCAAAATTAGACACAACATTGAAGATGGAAGC<br>GTTCAACTAGCAGACCATTATCAACAAAATACTCC<br>AATTGGCGATGGCCCTGTCCTTTTACCAGACAAC<br>CATTACCTGTCCACACAATCTGCCCTTTCGAAAG<br>ATCCCAACGAAAAGAGAGACCACATGGTCCTTCT<br>TGAGTTTGTAACAGCTGCTGGGATTACACATGGC<br>ATGGATGAACTATACAAA
rpoH P1 and P2 (σ70 and σS)	**T**<u>GCAATGGGTTCCGTAGCAGGGAAAGAGACCCC</u><br><u>GTTGTCTCTTCCCGGTATTTCATCTCTATGTCACA</u><br><u>TTTTGTGCGTAATTTATTCACAAGCTTGCATTGAA</u><br><u>CTTGTGGATAAAATCACGGTCTGATAAAACAGTG</u>

	AATGATAACCTCGTTGCTCTTAAGCTCTGGCACA<br>GTTGTTGCTACCACTGAAGCGCCAGAAGATATCG<br>ATTGAGAGGATTTGAATGACTGACAAAATGCAAA<br>GTTTAGCTTTAGCCCCAGTTGGCAACCTGGATTC<br>CTACATCCGGGCAGCTACTCGAGAGATCCTCTAG<br>ATTTAAGAAGGAGATATACATATGAGTAAAGGAGA<br>AGAACTTTTCACTGGAGTTGTCCCAATTCTTGTT<br>GAATTAGATGGTGATGTTAATGGGCACAAATTTTC<br>TGTCAGTGGAGAGGGTGAAGGTGATGCAACATA<br>CGGAAAACTTACCCTTAAATTTATTTGCACTACTG<br>GAAAACTACCTGTTCCATGGCCAACACTTGTCAC<br>TACTTTCGCGTATGGTCTTCAATGCTTTGCGAGA<br>TACCCAGATCATATGAAACAGCATGACTTTTTCAA<br>GAGTGCCATGCCCGAAGGTTATGTACAGGAAAG<br>AACTATATTTTTCAAAGATGACGGGAACTACAAGA<br>CACGTGCTGAAGTCAAGTTTGAAGGTGATACCCT<br>TGTTAATAGAATCGAGTTAAAAGGTATTGATTTTAA<br>AGAAGATGGAAACATTCTTGGACACAAATTGGAA<br>TACAACTATAACTCACACAATGTATACATCATGGC<br>AGACAAACAAAAGAATGGAATCAAAGTTAACTTC<br>AAAATTAGACACAACATTGAAGATGGAAGCGTTC<br>AACTAGCAGACCATTATCAACAAAATACTCCAATT<br>GGCGATGGCCCTGTCCTTTTACCAGACAACCATT<br>ACCTGTCCACACAATCTGCCCTTTCGAAAGATCC<br>CAACGAAAAGAGAGACCACATGGTCCTTCTTGA<br>GTTTGTAACAGCTGCTGGGATTACACATGGCATG<br>GATGAACTATACAAA
rpoH P3 (σ24)	**A**GTGAATGATAACCTCGTTGCTCTTAAGCTCTGG<br>CACAGTTGTTGCTACCACTGAAGCGCCAGAAGA<br>TATCGATTGAGAGGATTTGAATGACTGACAAAAT<br>GCAAAGTTTAGCTTTAGCCCCAGTTGGCAACCTG<br>GATTCCTACATCCGGGCAGCTACTCGAGAGATCC<br>TCTAGATTTAAGAAGGAGATATACATATGAGTAAA<br>GGAGAAGAACTTTTCACTGGAGTTGTCCCAATTC<br>TTGTTGAATTAGATGGTGATGTTAATGGGCACAAA<br>TTTTCTGTCAGTGGAGAGGGTGAAGGTGATGCA<br>ACATACGGAAAACTTACCCTTAAATTTATTTGCAC<br>TACTGGAAAACTACCTGTTCCATGGCCAACACTT<br>GTCACTACTTTCGCGTATGGTCTTCAATGCTTTG<br>CGAGATACCCAGATCATATGAAACAGCATGACTT<br>TTTCAAGAGTGCCATGCCCGAAGGTTATGTACAG<br>GAAAGAACTATATTTTTCAAAGATGACGGGAACTA

	CAAGACACGTGCTGAAGTCAAGTTTGAAGGTGA<br>TACCCTTGTTAATAGAATCGAGTTAAAAGGTATTG<br>ATTTTAAAGAAGATGGAAACATTCTTGGACACAAA<br>TTGGAATACAACTATAACTCACACAATGTATACAT<br>CATGGCAGACAAACAAAAGAATGGAATCAAAGTT<br>AACTTCAAAATTAGACACAACATTGAAGATGGAA<br>GCGTTCAACTAGCAGACCATTATCAACAAAATACT<br>CCAATTGGCGATGGCCCTGTCCTTTTACCAGACA<br>ACCATTACCTGTCCACACAATCTGCCCTTTCGAA<br>AGATCCCAACGAAAAGAGAGACCACATGGTCCT<br>TCTTGAGTTTGTAACAGCTGCTGGGATTACACAT<br>GGCATGGATGAACTATACAAA
rpoH P4 (σ70)	**G**ATAACCTCGTTGCTCTTAAGCTCTGGCACAGTT<br>GTTGCTACCACTGAAGCGCCAGAAGATATCGATT<br>GAGAGGATTTGAATGACTGACAAAATGCAAAGTT<br>TAGCTTTAGCCCCAGTTGGCAACCTGGATTCCTA<br>CATCCGGGCAGCTACTCGAGAGATCCTCTAGATT<br>TAAGAAGGAGATATACATATGAGTAAAGGAGAAG<br>AACTTTTCACTGGAGTTGTCCCAATTCTTGTTGA<br>ATTAGATGGTGATGTTAATGGGCACAAATTTTCTG<br>TCAGTGGAGAGGGTGAAGGTGATGCAACATACG<br>GAAAACTTACCCTTAAATTTATTTGCACTACTGGA<br>AAACTACCTGTTCCATGGCCAACACTTGTCACTA<br>CTTTCGCGTATGGTCTTCAATGCTTTGCGAGATA<br>CCCAGATCATATGAAACAGCATGACTTTTTCAAG<br>AGTGCCATGCCCGAAGGTTATGTACAGGAAAGA<br>ACTATATTTTTCAAAGATGACGGGAACTACAAGAC<br>ACGTGCTGAAGTCAAGTTTGAAGGTGATACCCTT<br>GTTAATAGAATCGAGTTAAAAGGTATTGATTTTAAA<br>GAAGATGGAAACATTCTTGGACACAAATTGGAAT<br>ACAACTATAACTCACACAATGTATACATCATGGCA<br>GACAAACAAAAGAATGGAATCAAAGTTAACTTCA<br>AAATTAGACACAACATTGAAGATGGAAGCGTTCA<br>ACTAGCAGACCATTATCAACAAAATACTCCAATTG<br>GCGATGGCCCTGTCCTTTTACCAGACAACCATTA<br>CCTGTCCACACAATCTGCCCTTTCGAAAGATCCC<br>AACGAAAAGAGAGACCACATGGTCCTTCTTGAG<br>TTTGTAACAGCTGCTGGGATTACACATGGCATGG<br>ATGAACTATACAAA
rpoH P5 (σ70)	**T**CTTAAGCTCTGGCACAGTTGTTGCTACCACTGA<br>AGCGCCAGAAGATATCGATTGAGAGGATTTGAAT<br>GACTGACAAAATGCAAAGTTTAGCTTTAGCCCCA

	GTTGGCAACCTGGATTCCTACATCCGGGCAGCT<br>ACTCGAGAGATCCTCTAGATTTAAGAAGGAGATA<br>TACATATGAGTAAAGGAGAAGAACTTTTCACTGG<br>AGTTGTCCCAATTCTTGTTGAATTAGATGGTGATG<br>TTAATGGGCACAAATTTTCTGTCAGTGGAGAGGG<br>TGAAGGTGATGCAACATACGGAAAACTTACCCTT<br>AAATTTATTTGCACTACTGGAAAACTACCTGTTCC<br>ATGGCCAACACTTGTCACTACTTTCGCGTATGGT<br>CTTCAATGCTTTGCGAGATACCCAGATCATATGAA<br>ACAGCATGACTTTTTCAAGAGTGCCATGCCCGAA<br>GGTTATGTACAGGAAAGAACTATATTTTTCAAAGA<br>TGACGGGAACTACAAGACACGTGCTGAAGTCAA<br>GTTTGAAGGTGATACCCTTGTTAATAGAATCGAGT<br>TAAAAGGTATTGATTTTAAAGAAGATGGAAACATT<br>CTTGGACACAAATTGGAATACAACTATAACTCACA<br>CAATGTATACATCATGGCAGACAAACAAAAGAAT<br>GGAATCAAAGTTAACTTCAAAATTAGACACAACAT<br>TGAAGATGGAAGCGTTCAACTAGCAGACCATTAT<br>CAACAAAATACTCCAATTGGCGATGGCCCTGTCC<br>TTTTACCAGACAACCATTACCTGTCCACACAATCT<br>GCCCTTTCGAAAGATCCCAACGAAAAGAGAGAC<br>CACATGGTCCTTCTTGAGTTTGTAACAGCTGCTG<br>GGATTACACATGGCATGGATGAACTATACAAA
rpoH P6 (σ54)	**C**GCCAGAAGATATCGATTGAGAGGATTTGAATGA<br>CTGACAAAATGCAAAGTTTAGCTTTAGCCCCAGT<br>TGGCAACCTGGATTCCTACATCCGGGCAGCTACT<br>CGAGAGATCCTCTAGATTTAAGAAGGAGATATAC<br>ATATGAGTAAAGGAGAAGAACTTTTCACTGGAGT<br>TGTCCCAATTCTTGTTGAATTAGATGGTGATGTTA<br>ATGGGCACAAATTTTCTGTCAGTGGAGAGGGTG<br>AAGGTGATGCAACATACGGAAAACTTACCCTTAA<br>ATTTATTTGCACTACTGGAAAACTACCTGTTCCAT<br>GGCCAACACTTGTCACTACTTTCGCGTATGGTCT<br>TCAATGCTTTGCGAGATACCCAGATCATATGAAAC<br>AGCATGACTTTTTCAAGAGTGCCATGCCCGAAG<br>GTTATGTACAGGAAAGAACTATATTTTTCAAAGAT<br>GACGGGAACTACAAGACACGTGCTGAAGTCAAG<br>TTTGAAGGTGATACCCTTGTTAATAGAATCGAGTT<br>AAAAGGTATTGATTTTAAAGAAGATGGAAACATTC<br>TTGGACACAAATTGGAATACAACTATAACTCACAC<br>AATGTATACATCATGGCAGACAAACAAAAGAATG<br>GAATCAAAGTTAACTTCAAAATTAGACACAACATT

	GAAGATGGAAGCGTTCAACTAGCAGACCATTATC<br>AACAAAATACTCCAATTGGCGATGGCCCTGTCCT<br>TTTACCAGACAACCATTACCTGTCCACACAATCT<br>GCCCTTTCGAAAGATCCCAACGAAAAGAGAGAC<br>CACATGGTCCTTCTTGAGTTTGTAACAGCTGCTG<br>GGATTACACATGGCATGGATGAACTATACAAA
P_{BAD}	AAACCAATTGTCCATATTGCATCAGACATTGCCGT<br>CACTGCGTCTTTTACTGGCTCTTCTCGCTAACCA<br>AACCGGTAACCCCGCTTATTAAAAGCATTCTGTAA<br>CAAAGCGGGACCAAAGCCATGACAAAAACGCGT<br>AACAAAAGTGTCTATAATCACGGCAGAAAAGTCC<br>ACATTGATTATTTGCACGGCGTCACACTTTGCTAT<br>GCCATAGCATTTTTATCCATAAGATTAGCGGATCC<br>TACCTGACGCTTTTTATCGCAACTCTCTACTGTTT<br>CTCC**A**<u>TACCCGTTTTTTTGGGCTAGCAGGAGGA</u><br><u>ATTCACC</u>ATGAGTAAAGGAGAAGAACTTTTCACT<br>GGAGTTGTCCCAATTCTTGTTGAATTAGATGGTG<br>ATGTTAATGGGCACAAATTTTCTGTCAGTGGAGA<br>GGGTGAAGGTGATGCAACATACGGAAAACTTAC<br>CCTTAAATTTATTTGCACTACTGGAAAACTACCTG<br>TTCCATGGCCAACACTTGTCACTACTTTCGCGTA<br>TGGTCTTCAATGCTTTGCGAGATACCCAGATCAT<br>ATGAAACAGCATGACTTTTTCAAGAGTGCCATGC<br>CCGAAGGTTATGTACAGGAAAGAACTATATTTTTC<br>AAAGATGACGGGAACTACAAGACACGTGCTGAA<br>GTCAAGTTTGAAGGTGATACCCTTGTTAATAGAAT<br>CGAGTTAAAAGGTATTGATTTTAAAGAAGATGGAA<br>ACATTCTTGGACACAAATTGGAATACAACTATAAC<br>TCACACAATGTATACATCATGGCAGACAAACAAAA<br>GAATGGAATCAAAGTTAACTTCAAAATTAGACACA<br>ACATTGAAGATGGAAGCGTTCAACTAGCAGACCA<br>TTATCAACAAAATACTCCAATTGGCGATGGCCCT<br>GTCCTTTTACCAGACAACCATTACCTGTCCACAC<br>AATCTGCCCTTTCGAAAGATCCCAACGAAAAGAG<br>AGACCACATGGTCCTTCTTGAGTTTGTAACAGCT<br>GCTGGGATTACACATGGCATGGATGAACTATACA<br>AA

Table S3: M9 Minimal Medium with glucose supplement Composition

Chemical	Concentration
Na_2HPO_4	0.048 M or 8.5 g/L
KH_2PO_4	0.022 M or 3 g/L
NH_4Cl	0.019 M or 1 g/L
NaCl	0.0037 M or 0.5 g/L
$CaCl_2$	0.0001 M or 0.0147 g/L
$ZnCl_2$	12.47 μM or 0.0017 g/L
$MgSO_4$	0.998 mM or 0.246 g/L
CAF (ammonium iron (III) citrate)	83 μM or 0.022 g/L
$MnCl_2$*4H_2O (trace elements)	5 μM or 0.001 g/L
$CuCl_2$*2H_2O (trace elements)	2.52 μM or 0.43 mg/L
$CoCl_2$*6H_2O (trace elements)	4.62 μM or 0.6 mg/L
Na_2MoO_4*2H_2O (trace elements)	2.91 μM or 0.6mg/L
glucose	0.4%

	MG1655 dnaK HS	MG1655 groEL HS	MG1655 rpoE HS	MG1655 rpoH HS	MG1655 dnaK PS	MG1655 groEL PS	MG1655 rpoE PS	MG1655 rpoH PS	AN62 dnaK HS	AN62 groEL HS	AN62 rpoE HS	AN62 rpoH HS	AN62 dnaK PS	AN62 groEL PS	AN62 rpoH PS
MG1655 dnaK HS		0.369619117	0.010408342	0.002171793	0.073456613	0.03476293	0.245866688	0.433506293	0.006236743	0.284927003	0.119731975	0.045492091	0.003325499	0.001736962	0.074440239
MG1655 groEL HS			0.006879067	0.000864574	0.049516094	0.028172209	0.309291174	0.234456095	0.002995562	0.125696044	0.137524043	0.038077222	0.001409974	0.00657751	0.035390606
MG1655 rpoE HS				0.06788671	0.006807433	0.111291861	0.021525822	0.002752181	0.43712235	0.002414521	0.131181616	0.069246668	0.163907915	0.000190572	0.00255515
MG1655 rpoH HS					0.002980502	0.010985311	0.003799975	0.000252566	0.047311625	0.000295334	0.033404477	0.006512771	0.188738172	5.83108E-05	0.000711568
MG1655 dnaK PS						0.013333797	0.041099551	0.0662202	0.005485786	0.092784066	0.029238729	0.015432285	0.003845963	0.05228587	0.299411471
MG1655 groEL PS							0.086277617	0.01013852	0.067228607	0.007826769	0.367263461	0.368787326	0.02282617	0.00308574	0.005969921
MG1655 rpoE PS								0.152412031	0.012913022	0.094576807	0.232743994	0.118770153	0.006222573	0.001037733	0.031773451
MG1655 rpoH PS									0.000869072	0.249845072	0.08058712	0.012646686	0.0040662	0.00055807	0.050906873
AN62 dnaK HS										0.000897578	0.105208313	0.037691958	0.146493704	0.000100626	0.001558626
AN62 groEL HS											0.058277341	0.009496545	0.000455486	0.000892632	0.092480828
AN62 rpoE HS												0.447537149	0.054397863	0.001811704	0.025285697
AN62 rpoH HS													0.013095835	0.000312419	0.006962103
AN62 dnaK PS														7.23745E-05	0.00977927
AN62 groEL PS															0.007426612
AN62 rpoH PS															

Table S4: Pairwise T test p values for comparisons between all promoters for all strains under all conditions (NS at 30°C, >HS at 42°C, NS at 37°C, and >PS at 60 MPa in both MG1655 and AN62). Green boxes indicate $p < 0.05$ and red boxes indicate $p >= 0.05$.

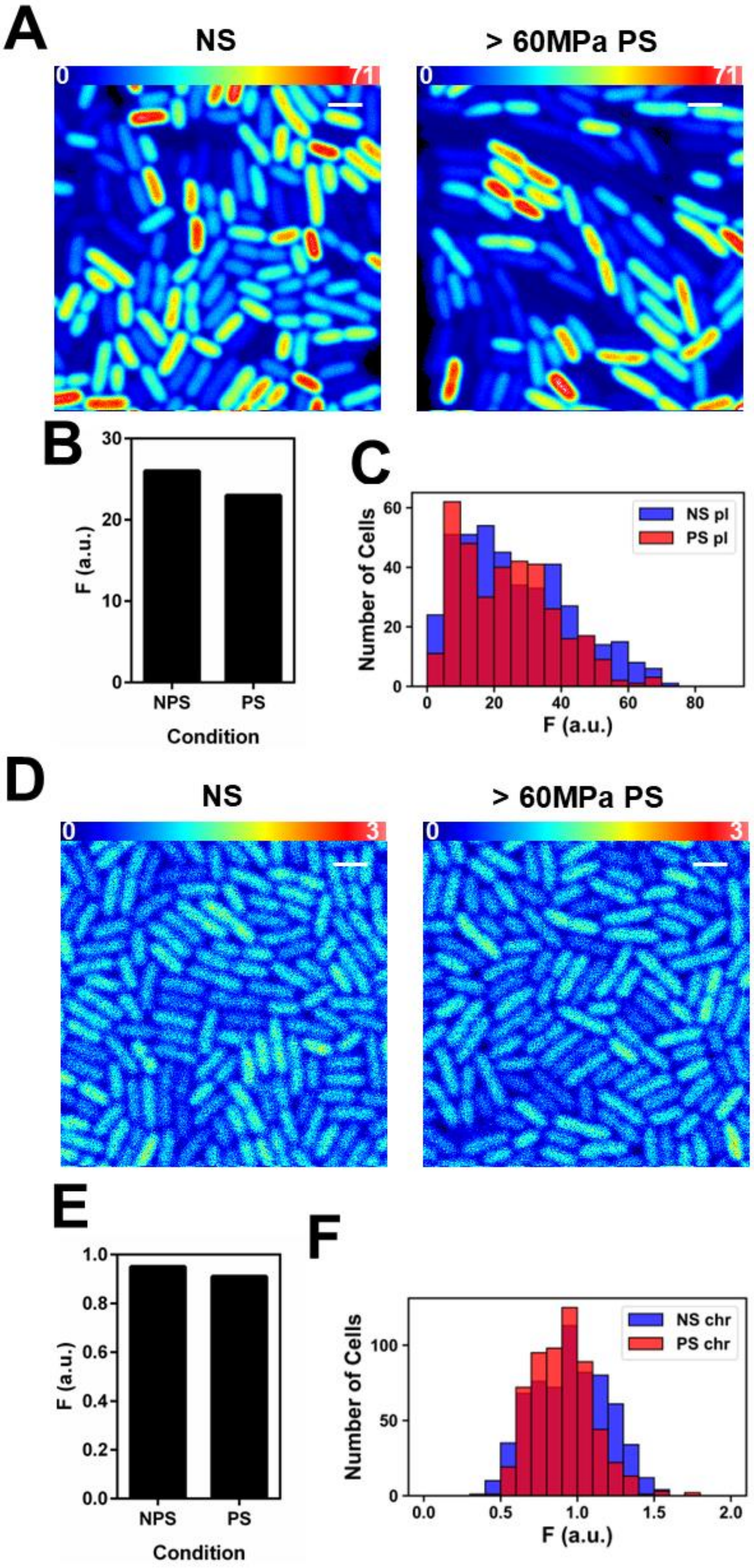
A
NS
> 60MPa PS
0
71
B
30
20
10
0
F (a.u.)
NPS
PS
Condition
C
Number of Cells
NS pI
PS pI
60
40
20
0
0
20
40
60
80
F (a.u.)
D
NS
> 60MPa PS
0
3
E
1.0
0.8
0.6
0.4
0.2
0.0
F (a.u.)
NPS
PS
Condition
F
Number of Cells
NS chr
PS chr
100
50
0
0.0
0.5
1.0
1.5
2.0
F (a.u.)

Figure S1. **Pressure has no effect on the expression of the non-heat shock P_{BAD} promoter**. (A) Representative sN&B images of MG1655 cells transformed with a multi-copy plasmid expressing GFP from the P_{BAD} promoter in presence of 0.002% arabinose before pressure shock (NS) and after pressure shock (PS). (B) Representative bar graphs representing average fluorescence intensity for all cells in 4 FOVs each before (NS) and after (PS) pressure shock. (C) Representative histogram distribution providing single cell resolution of the fluorescence intensities of cells that were and were not exposed to a 60MPa pressure shock. (D) Representative sN&B images of MG1655 cells expressing GFP from the P_{BAD} promoter in a chromosomal construct at the *mrr* locus (Bourges et al. 2017) in presence of 0.01 mM arabinose before pressure shock (NS) and after pressure shock (PS). (E) Bar graphs of average fluorescence intensity for all cells in 8 FOVs each before (NS) and after (PS) pressure shock. (F) Histograms of single cell intensities that were and were not exposed to a 60MPa pressure shock. The fluorescence intensity scale is posted above each image, and the length scale is the same between NS and PS images. Spatial scale bars (white) are 2 μm.
